# Genetic spectrum and characteristics of autosomal optic neuropathy in Korean: Use of next-generation sequencing in suspected hereditary optic atrophy

**DOI:** 10.3389/fneur.2022.978532

**Published:** 2022-08-22

**Authors:** Yuri Seo, Tae Young Kim, Dongju Won, Saeam Shin, Jong Rak Choi, Seung-Tae Lee, Byung Joo Lee, Hyun Taek Lim, Sueng-Han Han, Jinu Han

**Affiliations:** ^1^Department of Ophthalmology, Institute of Vision Research, Yongin Severance Hospital, Yonsei University College of Medicine, Yongin, South Korea; ^2^Department of Ophthalmology, Institute of Vision Research, Gangnam Severance Hospital, Yonsei University College of Medicine, Seoul, South Korea; ^3^Department of Laboratory Medicine, Yonsei University College of Medicine, Seoul, South Korea; ^4^Dxome Co., Ltd., Seongnam-si, South Korea; ^5^Department of Ophthalmology, Asan Medical Center, University of Ulsan College of Medicine, Seoul, South Korea; ^6^Seoul Orthopia Eye Clinic, Seoul, South Korea; ^7^Department of Ophthalmology, Institute of Vision Research, Severance Hospital, Yonsei University College of Medicine, Seoul, South Korea

**Keywords:** hereditary optic atrophy, inherited optic neuropathy, *OPA1*, *TMEM126A*, *SOX5*

## Abstract

**Aims:**

To evaluate the clinical characteristics and causative genetic variants in autosomal optic atrophy diagnosed using next-generation sequencing (NGS).

**Methods:**

A cohort of 57 unrelated families affected with bilateral optic atrophy were recruited from two university-based tertiary referral hospitals from May 2016 to April 2022. Genetic variants were detected using a target enrichment panel consisting of 429 or 595 genes and known deep intronic variants associated with inherited eye diseases, exome sequencing, or genome sequencing. The results of detailed clinical examinations, disease-causing variants, and clinical diagnoses were analyzed.

**Results:**

Among the 57 probands, 33 (57.9%) were men, and the median age at genetic testing was 19.1 years (interquartile range, 7.6–42.5 years). We identified 22 likely causative variants in 18 families and corresponding diagnostic yields of 31.6% (95% confidence interval, 21.0–44.5%). The diagnostic rate of NGS was higher in patients with infantile or early childhood onset optic atrophy than in those with late-onset or unknown optic atrophy (18/39, 46.2% vs. 0/18, 0%, *P* < 0.001). Among the 22 variants, 15 were novel in our cohort. The *OPA1* variants (*n* = 7) were found to be the major genetic causes, followed by the *NR2F1* variant (*n* = 4). The causative variants in *PTPN23, TMEM126A, NBAS*, and *WFS1* genes were identified in 4 probands with a recessive form of optic atrophy.

**Conclusions:**

Based on the results of diagnostic NGS for optic atrophy, the causative variant could be detected in 31.6% of patients. Our study also demonstrated that NGS is unlikely to help identify molecular causes in late-onset unexplained optic atrophy.

## Introduction

Inherited optic atrophy is the major underlying etiology of inherited visual impairment (1). This condition is the end result of injury to either the retinal ganglion cells, retinal nerve fiber layer, optic nerve, optic chiasm, or optic tract, and it is caused by various factors such as hereditary, metabolic, radiation-induced, malnutrition-induced, toxic, ischemic, inflammatory, or infiltrative lesions. In patients with optic atrophy, the pertinent history and ophthalmological findings (onset of symptoms, optic disc appearance, pattern of visual field loss, and color vision deficiency) may suggest specific causes of optic atrophy, but not all cases are readily classifiable (2). Defining the cause of optic atrophy in children is more difficult since complete ophthalmological assessments and recording clinical history are sometimes challenging.

To successfully diagnose this condition, first, a thorough gestational, prenatal, and neonatal history is essential because periventricular leukomalacia, intraventricular hemorrhage, or hydrocephalus can cause transsynaptic retrograde degeneration of the optic nerve (3). In addition, a previous history of head trauma, encephalitis, meningitis, malnutrition, or medications (e.g., vigabatrin and ethambutol) should be specifically elicited (2, 4). If no causes of optic atrophy are found in the history or clinical investigation, hereditary causes should be investigated. While dominant optic atrophy and Leber hereditary optic neuropathy (LHON) are the predominant forms of hereditary optic neuropathy (5), other rare diseases such as Bosch-Boonstra-Schaff optic atrophy syndrome (BBSOAS), *SSBP1* or *RTN4IP1* optic neuropathy, or Costeff syndrome should also be considered in the differential diagnosis (6). Patients with hereditary optic atrophy exhibit either isolated optic nerve dysfunction or accompanying syndromic features such as auditory, neurologic, or systemic abnormalities (7). Therefore, the recognition of accompanying signs and symptoms may aid in the diagnosis.

In recent years, next-generation sequencing (NGS) technology has transitioned from research to clinical use and is being used for inherited retinal diseases, congenital cataracts, and infantile nystagmus syndrome (8–10). Moreover, the use of genetic testing in diagnosis has been gaining more attention due to the recent studies on gene therapies for inherited optic neuropathies (11). Importantly, a few studies have also investigated the use of NGS on patients with hereditary optic atrophy (12–14). However, its clinical utility for diagnosing these patients remains largely unknown. Therefore, herein, the genotypic and phenotypic characteristics of optic atrophy diagnosed with NGS were analyzed in patients with suspected hereditary optic atrophy over the past 6 years in two tertiary referral centers.

## Materials and methods

### Recruitment and selection of patients with hereditary optic atrophy

The study cohort consisted of 57 unrelated, consecutively sampled patients with suspected hereditary optic atrophy who were undergoing further investigation and were recruited from two university-based tertiary referral hospitals: the Asan Medical Center, Seoul, South Korea and Severance Hospital, Seoul, South Korea between March 2016 and March 2022. All patients had an insidious onset of optic atrophy. Patients who were previously diagnosed with known neurodegenerative disorders commonly associated with optic atrophy such as Leigh syndrome; myoclonic epilepsy with ragged-red fibers; mitochondrial myopathy, encephalopathy, lactic acidosis, and stroke-like episodes; neuropathy, ataxia, and retinitis pigmentosa; cerebellar ataxia, areflexia, pes cavus, optic atrophy, and sensorineural hearing loss syndrome; Canavan disease; Charcot-Marie-Tooth disease; metachromatic leukodystrophy; metabolic syndromes such as 3-methylglutaconic aciduria or maple syrup urine disease; or Pelizaeus-Merzbacher disease were excluded from the study. Careful history-taking was done to exclude patients with risk factors for any non-genetic causes of optic atrophy such as a history of prematurity (e.g., periventricular leukomalacia and hypoxic ischemic encephalopathy), severe malnutrition, infectious diseases, a previous history of cancer, demyelinating diseases, non-arteritic anterior ischemic optic neuropathy, head trauma, or drug-induced or toxic optic neuropathy (e.g., ethambutol, linezolid or heavy metals).

In this study, all the patients had an insidious onset of bilateral generalized or temporal optic atrophy, and none of the patients displayed band optic atrophy or superior segmental optic atrophy. Sanger sequencing for mitochondrial *ND1, ND4*, and *ND6* genes associated with LHON was also previously conducted in all patients except those with congenital or infantile-onset optic atrophy. No structural abnormalities explaining the bilateral optic atrophy were identified in the previous brain magnetic resonance imaging (MRI) or computerized tomography scans.

All patients underwent ophthalmologic examination, which included the measurement of visual acuity and slit-lamp and fundus examinations. If applicable, a color vision test, spectral domain optical coherence tomography, and automated visual field tests were also performed. Peripheral blood samples from the patients were also collected for genetic analysis. Informed written consents were obtained from all the patients. This study was approved by the institutional review board of Gangnam Severance Hospital, Seoul, South Korea (3-2020-0063) and adhered to the tenets of the Declaration of Helsinki.

### NGS analysis

NGS analysis was primarily performed using the customized NGS panel analysis with 429 or 595 targeted genes (Supplementary Tables 1–3) or exome sequencing (ES). Secondarily, exome sequencing (ES) or genome sequencing (GS) were proposed as a diagnostic option for patients when targeted panel sequencing failed to identify the causative variants. With the consent of the patients, target enrichment for targeted panel was performed with a molecular inversion probe-based capture method using a customized target enrichment kit (Dxome, South Korea). ES was performed using either the xGen Exome Research Panel v1 (Integrated DNA technologies, Coralville, IA, USA) or Twist Human Core Exome kit (Twist Bioscience, San Francisco, CA, USA). If the identification was unsuccessful following ES, GS was suggested using the TruSeq Nano DNA sample prep kit (Illumina, San Diego, CA, USA). Pooled libraries were sequenced using NextSeq 550 for the targeted sequencing panel and NovaSeq6000 (Illumina, San Diego, CA, USA) for ES or GS. NGS data analysis was performed primarily through our custom pipeline (9, 15). The interpretation of variants was done according to the 5-tier classification system recommended by the American College of Medical Genetics and Genomics and the Association for Molecular Pathology using a step-by-step approach (Supplementary Figure 1) (16). The systematic approaches for variant classification have also been described in Supplementary Methods.

## Results

### Clinical characteristics

Among the 57 unrelated Korean patients, 33 patients (57.9%) were male, and the median age of the patients at the time of genetic testing was 19.1 years (interquartile range [IQR], 7.6–42.5 years, Supplementary Figure 2 in the Supplement). The median best-corrected visual acuity was 0.30 (IQR, 0.15–0.56) in the right eye and 0.40 (IQR, 0.13–0.85) in the left eye (logMAR). Seventeen patients (29.8%) had systemic features such as facial dysmorphism, intellectual disability, juvenile-onset diabetes mellitus, cerebellar atrophy, or developmental delay, while the remaining forty patients only had non-syndromic isolated dominant optic atrophy. The onset of optic atrophy was either infantile (*n* = 14), early childhood (*n* = 25), late-onset (*n* = 13), or unknown (*n* = 5). Seven probands had family histories of optic atrophy. Among these, dominant inheritance was noted in Six patients and autosomal recessive pattern of inheritance in one patient. Sixteen patients (28.1%) had various types of nystagmus including infantile, manifest latent, gaze-evoked, or spasmus nutans-like nystagmus. The clinical features of the patients are summarized in Table 1 and Supplementary Table 4.

**Table 1:** The clinical features of 18 patients with hereditary optic atrophy and their families.

**Pt**	**Initial diagnosis**	**Sequencing method**	**Moleculardiagnosis**	**Final diganosis**	**Sex**	**Age** **(y)**	**Onset** **age**	**Nystagmus**	**Refraction**		**BCVA (OD/OS) logMAR**	**Fundus**	**Average RNFL thickness^c^ (temporal, μm)**		**ERG**	**Additional phenotypes**
									**OD**	**OS**			**OD**	**OS**		
1	DOA	Targeted panel	*OPA1*	DOA	F	47	Early childhood onset	None	−7.25	−6.25	0.40/0.52	Temporal optic atrophy	61 (38)	62 (39)	Normal	None
1-1^b^	DOA		Not tested	DOA	F	49	Early childhood onset	None	−11.5	−10.75	0.52/0.62	Generalized optic atrophy	58	58	NA	None
2	DOA	Targeted panel	*OPA1*	DOA	M	6.5	Early childhood onset	Multidirectional nystagmus	1	1.5	1.70/1.30	Generalized optic atrophy	NA	NA	Normal	None
3^a^	DOA	Targeted panel	*OPA1*	DOA	M	9	Early childhood onset	None	0	−1	0.40/0.30	Temporal optic atrophy	85 (44)	86 (45)	NA	None
4	DOA	Targeted panel	*OPA1*	DOA	M	5	Early childhood onset	None	0.5	0.75	0.52/1.00	Temporal optic atrophy	63 (46)	53 (41)	NA	None
5	DOA	Targeted panel	*OPA1*	DOA	M	7.6	Early childhood onset	None	−0.5	−0.25	0.30/0.30	Temporal optic atrophy	55 (34)	57 (35)	NA	None
6	DOA	Targeted panel	*OPA1*	DOA	M	6.3	Early childhood onset	None	−1	0.375	0.22/0.22	Temporal optic atrophy	91 (33)	92 (32)	NA	None
6-1^b^	DOA	Sanger	*OPA1*	DOA	M	38	Early childhood onset	None	NA	NA	0.09/0.3	NA	NA	NA	NA	None
7	DOA	ES	*OPA1*	DOA	F	15.1	Early childhood onset	None	−1.125	−1.50	0.79/0.69	Temporal optic atrophy	58 (30)	60 (30)	NA	None
8^a^	DOA	Targeted panel	*NR2F1*	BBSOAS	M	6.6	Infantile onset	Latent nystagmus	−3.25	−2.25	0.70/0.70	Generalized optic atrophy	NA	46	Normal	Delayed development, intellectual disability, micrognathia
9^a^	BBSOAS	Targeted panel	*NR2F1*	BBSOAS	M	19.1	Infantile onset	Latent nystagmus	0.75	1.25	0.05/0.70	Generalized optic atrophy	47	NA	NA	Delayed development
10^a^	BBSOAS	ES	*NR2F1*	BBSOAS	F	19.2	Infantile onset	Latent nystagmus	−2.5	−2	1.70/1.30	Generalized optic atrophy	NA	NA	NA	Delayed development, intellectual disability, facial dysmorphism
11	DOA	Targeted panel	*NR2F1*	BBSOAS	M	25.8	Infantile onset	Latent nystagmus	−2.5	−2.75	0.40/0.52	Generalized optic atrophy	52	54	NA	Delayed development, speech delay
12^a^	Unknown cause	Targeted panel	*SOX5*	Lamb-Shaffer syndrome	F	8.1	Infantile onset	None	−2	−2.25	0.22/0.40	Generalized optic atrophy	63	59	NA	Facial dysmorphism, intellectual disability
13	DOA	ES	*SPG7*	*SPG7*-associated optic atrophy	M	15.6	Infantile onset	Infantile nystagmus	−4.0	−5.0	0.30/0.52	Generalized optic atrophy	44	41	Normal	None
14^a^	Unknown cause	ES	*NBAS*	SOPH syndrome	M	28.1	Early childhood onset	None	−1.5	−1.25	0.60/0.40	Generalized optic atrophy, Cone dystrophy	41	38	Decreased light adapted response	Short stature, senile face, history of frequent upper respiratory infections
15^a^	BBSOAS	Targeted panel/ ES	*PTPN23*	*PTPN23* optic atrophy	F	5.8	Early childhood onset	Spasmus nutans-like nystagmus	−2.75	−3.75	0.52/0.52	Generalized optic atrophy	48	43	Normal	Hypotonia, delayed development
16	BBSOAS	Targeted panel	*TMEM126A*	*TMEM126A* optic atrophy	M	6.6	Early childhood onset	Latent nystagmus	0.25	0.375	1.70/1.40	Generalized optic atrophy	42	36	NA	None
17	Wolfram syndrome	Targeted panel	*WFS1*	Wolfram syndrome	F	7.2	Early childhood onset	None	0.25	−0.25	1.40/1.40	Generalized optic atrophy	49	53	NA	Type I DM, diabetes insipidus
17-1^b^	Wolfram syndrome	Sanger	*WFS1*	Wolfram syndrome	M	4.9	Early childhood onset	None	0	−0.38	0.52/0.39	Generalized optic atrophy	79	93	NA	Type I DM
18	DOA	ES/GS	*SSBP1*	*SSBP1* dominant optic atrophy	M	35.4	Early childhood onset	None	−0.375	−1	1.39/1.39	Generalized optic atrophy	31	37	Normal	None
18-1 ^b^	NA	Sanger	*SSBP1*	*SSBP1* dominant optic atrophy	F	57.4	Early childhood onset	None	0.75	0.5	1.39/1.39	Generalized optic atrophy	41	45	NA	None

BBSOAS, Bosch-Boonstra-Schaff Optic Atrophy Syndrome; ES, Exome sequencing; GS, Genome sequencing; Targeted panel/ES, Targeted panel sequencing followed by ES; ES/GS, Exome sequencing followed by GS; BCVA, Best Corrected Visual Acuity; CSNB, Congenital Stationary Night Blindness; DM, Diabetes mellitus; DOA, Dominant Optic Atrophy; F, Female; M, Male; NA, Not Available; RNFL, retinal nerve fiber layer; UCSM, no constant, no steady, no maintained fixation; HC, Head circumference; OD, right; OS, left; SOPH, Short stature with Optic atrophy and Pelger-Huet anomaly; Infantile onset ≤ 1 year old; Early childhood onset ≤ 13 year old; Late onset ≥13 years old.

a Novel, but previously reported by the authors.

b The families of patients. P1-1 indicates the sister of P1. P6-1 indicates the father of P6. P17-1 indicates the younger brother of P17. P18-1 indicates the mother of P18.

C In the case with temporal optic atrophy, temporal RNFL thickness was revealed in parenthesis below the average thickness.

### NGS results and genetic findings

**Figure 1:**
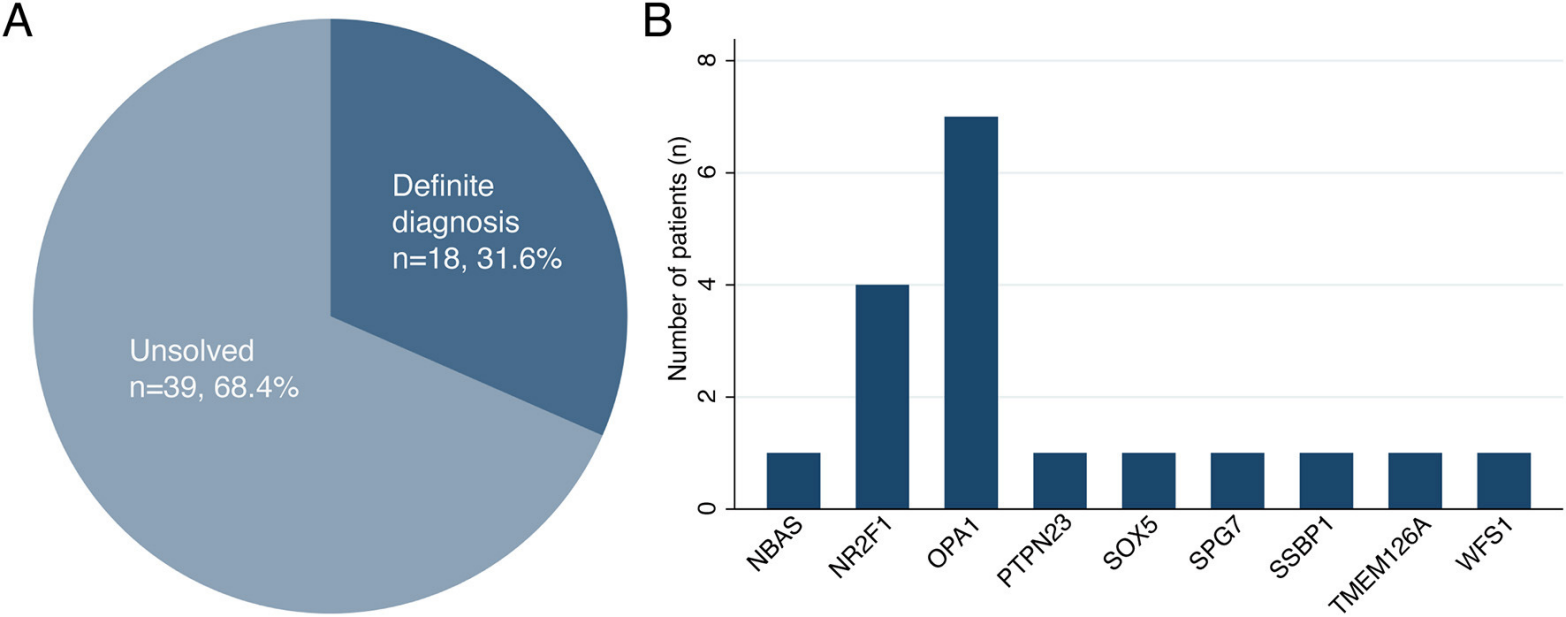
**(A,B)** The diagnostic rate of next-generation sequencing and genetic distribution of causative variants with hereditary optic atrophy.

A total of 18 patients received molecular diagnoses after NGS, while 39 patients remained as unsolved cases, corresponding to a molecular detection rate of 31.6% (95% confidence interval, 21.0–44.5%) (Figure 1). Among the 18 solved cases, 12 cases (66.7%) were diagnosed using targeted panel sequencing and the remaining 6 cases (33.3%) were solved using ES only (*n* = 4), targeted panel sequencing followed by ES (*n* = 1) or ES followed by GS (*n* = 1). For the 39 patients with unsolved cases, either targeted panel sequencing (*n* = 32) or ES (*n* = 7) was performed without further testing.

Furthermore, a total of 22 disease-causing variants were identified, 15 of which were novel (Table 2; Supplementary Table 5). Among the 39 patients with unsolved cases, one patient was found to have only one likely pathogenic variant in his/her recessive genes (Supplementary Table 6; Supplementary Figure 3. The causative variants were identified in all seven out of the seven patients with a familial history of optic neuropathy (100%), a diagnostic rate higher than in sporadic cases (22.0%) (*P* < 0.001, Fisher's exact test). Moreover, there was no difference in the diagnostic rate of NGS between the syndromic and non-syndromic cases (47.1 vs. 25.0%, *P* = 0.101). There was no significant difference in the diagnosis rate according to the presence or absence of nystagmus (43.8 vs. 26.8%, *P* = 0.217). The diagnostic rate of NGS was much higher in the patients with infantile or early childhood onset optic atrophy than in the patients with late-onset or unknown optic atrophy (18/39, 46.2 vs. 0/18, 0%, *P* < 0.001).

**Table 2:** Likely causative variants identified in patients with hereditary optic atrophy.

**Pt**	**Gene**	**Variants**	**Zygosity**	**Segregation**	**gnomAD MAF**	**Exon (Intron)**	**Previous literature (PMID)**	**Domain**	**ACMG**	**Accession ID** **for transcript**
1	*OPA1*	c.2297dupT:p.(Met766Ilefs*25)	Hetero	NA	Not found	23/29	Novel	Dynamin domain	P	NM_015560.2
2	*OPA1*	c.1240A>C:p.(Thr414Pro)	Hetero	De novo	Not found	13/29	26905822	GTPase domain	P	NM_015560.2
3^a^	*OPA1*	c.795_798del:p.(Asp266Cysfs*41)	Hetero	NA	Not found	8/29	35052368^b^, ^c^	GTPase domain	P	NM_015560.2
4	*OPA1*	c.305A>G:p.(Tyr102Cys)	Hetero	Maternal	5/248904	2/29	19319978	-	LP	NM_015560.2
5	*OPA1*	c.1202G>A:p.(Gly401Asp)	Hetero	NA	Not found	12/29	17029191	GTPase domain	LP	NM_015560.2
6	*OPA1*	c.784A>T:p.(Lys262*)	Hetero	Paternal	Not found	8/29	Novel	GTPase domain	LP	NM_015560.2
7	*OPA1*	c.1620_1622del:p.(Thr541del)	Hetero	NA	Not found	18/30	22042570	Dynamin domain	LP	NM_015560.2
8^a^	*NR2F1*	c.513C>G:p.(Tyr171*)	Hetero	NA	Not found	2/3	31393201^b^	Between DBD-LBD	LP	NM_005654.4
9^a^	*NR2F1*	c.91_93dupCGC:p.(Arg31dup)	Hetero	NA	Not found	1/3	35052368^b^	DNA binding domain	LP	NM_005654.4
10^a^	*NR2F1*	c.51_69dup:p.(Asn24Glyfs*379)	Hetero	NA	Not found	1/3	35052368^b^	DNA binding domain	P	NM_005654.4
11	*NR2F1*	c.1080C>A: p.(Tyr360*)	Hetero	NA	Not found	3/3	Novel	Ligand binding domain	P	NM_005654.4
12^a^	*SOX5*	Whole gene deletion	Hetero	NA	Not found	-	35052368^b^	-	P	NM_001261414.2
13	*SPG7*	c.1224T>G:p.(Asp408Glu)	Hetero	De novo	Not found	9/17	Novel	AAA protease domain	LP	NM_003119.3
14^a^	*NBAS*	c.3494del:p.(Val1165Serfs*31) c.5740C>T:p.(Arg1914Cys)	Compound hetero	Maternal Paternal	Not found Not found	30/52 45/52	34110364^b^ 34110364^b^	Secretory pathway Sec39 -	LP LP	NM_015909.3
15^a^	*PTPN23*	c.3768del:p.(Pro1258Argfs*2) c.4886C>G:p.(Pro1629Arg)	Compound hetero	Paternal Maternal	Not found Not found	20/25 25/25	35427297^b^ 35427297^b^	Protein-tyrosine phosphatase -	US LP	NM_015466.3
16	*TMEM126A*	c.28del:p.(Glu10Lysfs*3) c.163C>T:p.(Arg55*)	Compound hetero	Paternal Maternal	3/249618 8/251482	2/5 3/5	Novel 19327736	DUF1370 DUF1370	LP LP	NM_032273.3
17	*WFS1*	c.631+1del c.2262_2263del:p.(Cys755Serfs*3)	Compound hetero	NA Maternal	Not found Not found	(5/7) 8/8	Novel 28432734	- -	LP LP	NM_006005.3
18	*SSBP1*	c.364A>G: p.(Lys122Glu)	Hetero	Maternal	Not found	6/7	35946466*^b^*	SSB domain	LP	NM_001256510.1

DBD, DNA binding domain; gnomAD, genome aggregation database; LBD, ligand binding domain; SSB, single stranded binding; LP, likely pathogenic; MAF, minor allele frequency; NA, not available; P, pathogenic; US, uncertain significance; gnomAD v2.1.1 was used for calculating the minor allele frequency of the variant. ACMG/AMP guideline: The American College of Medical Genetics and Genomics (ACMG) and the Association for Molecular Pathology (AMP) 2015 updated standards and guidelines for the clinical interpretation of sequence variants.

a These variants were previously reported patients by the authors.

b Novel, but previously reported by the authors. (Report for P18 is in press).

C This variant is novel, but similar deletion with same amino acid change was reported in c.796_799delGACA:p.(Asp266Cysfs*41) in Yu-Wai-Man (2011) Ophthalmology.

The most frequently mutated genes were *OPA1* (*n* = 7) and *NR2F1* (*n* = 4). The variants in *SOX5* (*n* = 1)*, SPG7* (*n* = 1), and *SSBP1* (*n* = 1) genes were responsible for cases of other dominantly inherited optic atrophies. Among the cases of recessive optic atrophy, there was one case of short stature, optic nerve atrophy, Pelger-Huet anomaly (SOPH syndrome) caused by *NBAS* variants, one case of *PTPN23* optic atrophy syndrome, one case of *TMEM126A* optic atrophy, and one case of Wolfram syndrome. The clinical phenotypes and genotype results of the 18 patients with definite diagnoses are summarized in Tables 1, 2. The initial clinical diagnoses were revised in eight patients (14%) after performing NGS.

### Genotype-phenotype correlations

#### OPA1 dominant optic atrophy

All seven cases of optic atrophy with variants in *OPA1* were non-syndromic. P2 exhibited a visual acuity of 1.70 in the right eye and 1.30 in the left eye (logMAR). Furthermore, optical coherence tomography imaging confirmed the presence of severe thinning of the peripapillary retinal nerve fiber layer in all quadrants (Supplementary Figure 4). This patient had been diagnosed with multidirectional nystagmus at 17 months of age. Targeted NGS on this patient revealed a de novo c.1240A>C:p.(Thr414Pro) variant in the *OPA1* gene. A previous study reported of a patient with the same variant who had a visual acuity of 1.52 in the right eye and 2.0 in the left eye (logMAR) (17). Notably, missense *OPA1* variants located within the GTPase catalytic domain are more likely to cause severe phenotypes than variants resulting in haploinsufficiency (6, 18).

#### Lamb–Shaffer syndrome

**Figure 2:**
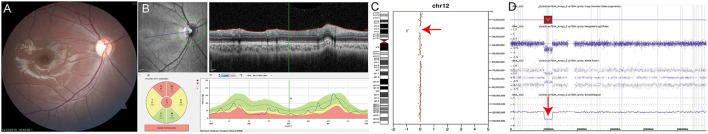
(P12) Chromosomal copy number variations analysis using off-target reads reveals *SOX5* deletion in Lamb–Shaffer syndrome. An 8-year-old female patient shows strabismus and hirsutism in the philtrum area. Best corrected visual acuity is 0.22 in the right eye and 0.40 in the left eye (logMAR). **(A,B)** Fundus photography and spectral-domain optical coherence tomography show optic nerve atrophy in both eyes (right eye was shown). **(C)** Deletion of the chromosome 12p12 is suspected by bioinformatics analysis using CopywriteR program (red arrow). **(D)** Array comparative genomic hybridization confirms 12p12.2p12.1 deletion (red arrow). Optic atrophy is thought to be related to *SOX5* gene deletion. Regular cardiac function check-up is recommended because *ABCC9* gene deletion is found by array comparative genomic hybridization.

P12 was an 8-year-old female patient presenting with dissociated vertical deviation and inferior oblique muscle overaction. On fundus examination, fundus photography and spectral-domain optical coherence tomography showed bilateral diffuse optic atrophy in both eyes (Figures 2A,B, right eye is shown). Visual acuity was 0.22 in the right eye and 0.40 in the left eye (logMAR). P12 exhibited no definite delayed development or nystagmus, but mild facial dysmorphic features and hirsutism in the philtrum area were noted. Deletion of chromosome 12p12 was suspected based on bioinformatics analysis using CopywriteR program (Figure 2C, red arrow), and array comparative genomic hybridization confirmed 12p12.2p12.1 deletion (hg19:chr12:20286266-25154015, Figure 2D, red arrow). Optic atrophy was considered as a clinical feature of *SOX5* gene deletion, known as Lamb–Shaffer syndrome. This chromosomal deletion encompasses several disease-associated genes such as *ABCC9, GYS2, LDHB, PDE3A, PYROXD1, SLCO1B1, SLCO1B3*, and *SOX5*. Among these gene, only *ABCC9, PDE3A*, and *SOX5* were inherited as autosomal dominant. In dosage sensitivity curation in ClinGen, only *SOX5* had sufficient evidence of haploinsufficiency score: 3. The other genes were not determined yet. All reported variants in *PDE3A* gene, which causes hypertension and brachydactyly syndrome, were missense variants, and blood pressure, fingers and toes were normal. Because pathogenic variants in *ABCC9* gene had been known to cause dilated cardiomyopathy or atrial fibrillation, regular cardiac function check-up was recommended for the patient.

#### *TMEM126* optic atrophy

**Figure 3:**
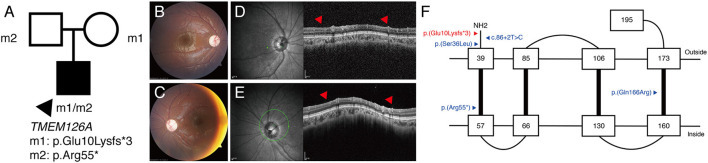
(P16) *TMEM126A* optic atrophy. The best-corrected visual acuity is 1.70 in the right eye and 1.40 in the left eye (logMAR). **(A)** Segregation analysis revealing the presence of compound heterozygous *TMEM126A* variants. **(B,C)** Fundus photographs showing diffuse optic atrophy **(D,E)** Profound loss of peripapillary retinal nerve fiber layer is detected in both eyes (red arrowhead). **(F)** Schematic representation of the *TMEM126A* protein and previously reported variants (blue). The red color indicates the novel variant in this study.

P16 was a 6-year-old male patient with bilateral optic atrophy and low visual acuity. His best corrected visual acuity was 1.70 in the right eye and 1.40 in the left eye (logMAR). He had a mild degree of delayed development and latent nystagmus, but had normal intelligence. The patient's initial clinical diagnosis was BBSOAS. The targeted NGS showed the presence of the novel compound heterozygous *TMEM126A* c.28del:p.(Glu10Lysfs^*^3) / c.163C>T:p.(Arg55^*^) variants (Figure 3A). Based on this, the patient's clinical diagnosis was revised to *TMEM126A* optic atrophy. Diffuse optic atrophy was observed on fundus photography (Figures 3B,C), and profound loss of peripapillary retinal nerve fiber layer was detected on spectral-domain optical coherence tomography (Figures 3D,E). The location of this novel variant is indicated in a schematic representation of the *TMEM126A* protein comparing previously reported variants (Figure 3F).

#### Wolfram syndrome

**Figure 4:**
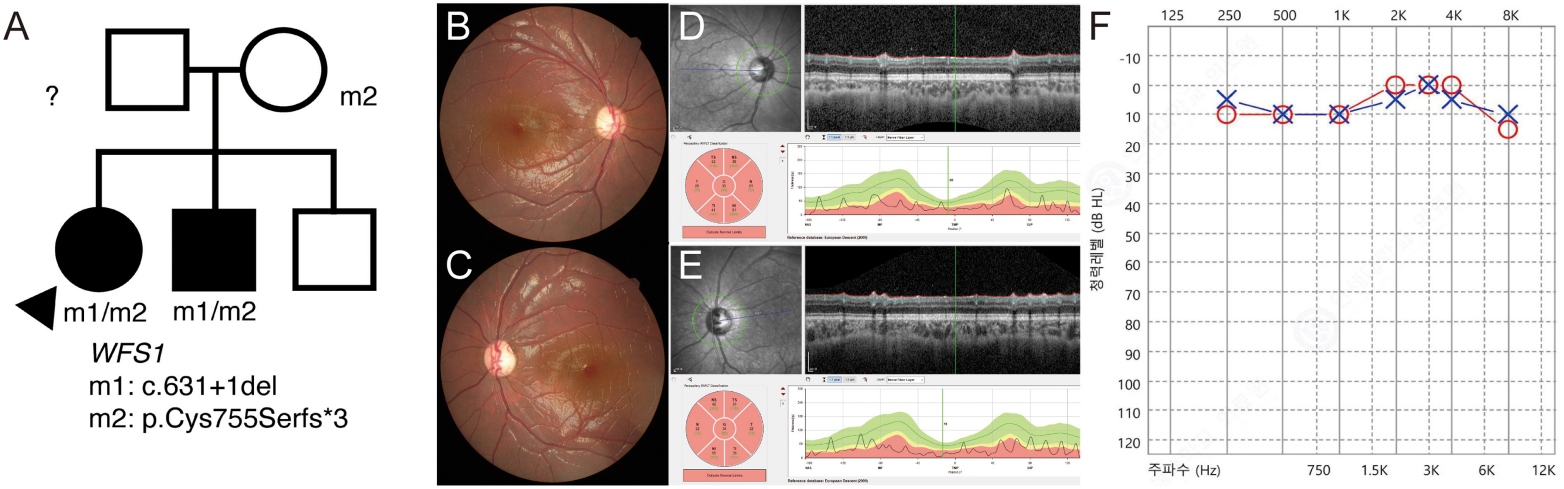
(P17) Targeted next-generation sequencing identify *WFS1* variants in optic atrophy with diabetic ketoacidosis. Clinical diagnosis before genetic testing is Wolfram syndrome. Best corrected visual acuity is 1.40 logMAR in both eyes. **(A)** The segregation analysis shows compound heterozygous c.631+1del/c.2262_2263del:p.(Cys755Serfs*3) variants. These variants are also detected in her brother. The paternal sample is not available. **(B,C)** Fundus photographs showing generalized diffuse optic atrophy. **(D,E)** The optical coherence tomography revealing generalized retinal nerve thinning. **(F)** Audiometry is normal at the age of 7 years.

P17 was a 7-year-old female patient who was brought to the emergency room with a complaint of severe fatigue. Examination revealed diabetic ketoacidosis, which was her first diagnosis of diabetes. She was diagnosed with optic atrophy 2 years ago and had never been genetically evaluated. Her best corrected visual acuity was 1.4 in both eyes (logMAR) and showed no definite nystagmus. She was diagnosed with type 1 diabetes mellitus, but no definite developmental delay or intellectual disability was noted. The segregation analysis revealed compound heterozygous c.631+1del/c.2262_2263del:p.(Cys755Serfs3) variants. Therefore, she was diagnosed with Wolfram syndrome. These variants were also found in her brother (Figure 4A). The c.631+1del variant was identified as a novel variant (Supplementary Figure 5). Generalized optic atrophy was shown on fundus photography and optical coherence tomography (Figures 4B–E). An audiometry test showed normal hearing function (Figure 4F).

The clinical features of the BBSOAS in P11 is presented in Supplementary Figure 6. In early childhood, P11 presented delayed speech that was corrected with rehabilitation. At the age of 25-years old, P11 revealed normal intelligence and no other neurological symptoms. Fundus photographs revealed diffuse RNFL thinning. BCVA was 0.4 in the right eye and 0.52 in the left eye (logMAR). Targeted sequencing identified c.1080C>A:p.(Tyr360^*^) variant in *NR2F1*.

The clinical information of SOPH syndrome and *PTPN23* optic atrophy were described in our previous study (19, 20). P8, P9, and P10 were reported in our previous study (1, 21).

## Discussion

The molecular diagnostic yield for hereditary optic atrophy is relatively low compared to that for other hereditary eye diseases such as inherited retinal dystrophy or infantile nystagmus syndrome (diagnostic rate: 75–90%) (1, 9). Previous studies reported various diagnostic rates for hereditary optic atrophy ranging from 20.2–40% (12–14). Our study demonstrated that NGS identified the causative variants in 31.6% of the patients with suspected hereditary optic atrophy. This result is comparable to that of previous studies. In addition, variants in *OPA1* were observed to be major causes of optic atrophy in our cohort, accounting for 38.9% of our solved cases. BBSOAS was also found to be a common cause of optic atrophy, accounting for 22.2% of our solved cases. A previous study reported that 78.9% of solved cases in their study were caused mainly by *OPA1* gene variants followed by *WFS1* gene variants (12). Yu-Wai-Man et al. reported that screening the *OPA1* and *OPA3* genes allowed for the detection of pathogenic variants in 27 (14.4%) of the 188 probands in their study, a finding which is consistent with our study (22). No *OPA3* variants were found in our cohort.

Unexplained insidious onset of optic atrophy in adulthood might result from genetic defects. A previous study reported that the age of onset of isolated optic atrophy was 20–50 years in *AFG3L2* and *SPG7* dominant optic atrophy. However, our study failed to identify the responsible genetic causes for unexplained adult-onset optic atrophies. This can be interpreted in various ways. A recent study found dominant *MEIF1* variants as a cause for late-onset optic neuropathy in two unrelated patients (23). Therefore, other novel genes, including *MEIF1*, might be the cause of late-onset optic neuropathy in our cohort. Second, non-coding pathogenic variants or structural variants can be missed, and those were misclassified as unsolved. Third, unknown cause of late-onset optic atrophy can be resulted from toxic, medication-induced, or environmental causes which patients did not recognize previously.

The *SOX5* gene encodes the member of the SOX (SRY-related high mobility group-box) gene family that is related to the regulation of embryonic development such as neurogenesis and skeletogenesis (24). Haploinsufficiency of *SOX5* causes Lamb–Shaffer syndrome, clinically characterized with developmental delay, speech delay, and behavioral disturbances (25). Ophthalmic features, such as strabismus, optic nerve atrophy, amblyopia, and cortical visual impairment have been frequently reported (26, 27). In our patient, mild intellectual disability and facial dysmorphism was noted, which have been known as the most common symptoms of Lamb–Shaffer syndrome (28). The large deletion also included *ABCC9* gene, and pathogenic variant in this gene is known to cause cardiomyopathy, excess hair growth, and intellectual disability named as Cantú syndrome. Most reported pathogenic variant in *ABCC9* gene was missense, and null variants had been reported to be associated with dilated cardiomyopathy. Therefore, regular monitoring of cardiac function is needed in this patient.

*TMEM126A* is a gene that encodes an assembly factor for the ND4-module of mitochondrial complex I (29, 30), in which variants of this gene cause non-syndromic autosomal-recessive optic atrophy (31). The TMEM126A protein is located in the mitochondrial cristae, along with the OPA1 protein. Notably, our case is the very first case of *TMEM126A* optic atrophy reported in East Asian patients. The c.163C>T/p:(Arg55^*^) variant has been well known as a founder variant in North African descent (31, 32). The minor allele frequencies of the novel c.28del:p.(Glu10Lysfs^*^3) variant were reported to be 3/18358 in East Asians in the genome aggregation database (gnomAD) and 0.0002 in the 4.7K ToMMo (Tohoku Medical Megabank Organization) Japanese database (33). Therefore, we expect more cases with *TMEM126A* optic atrophy to be discovered among East Asian patients.

Wolfram syndrome has clinically been termed as “DIDMOAD” (diabetes insipidus, diabetes mellitus, optic atrophy, and deafness), and is usually inherited with an autosomal recessive pattern. *WFS1* encodes wolframin which is a transmembrane protein localized to the endoplasmic reticulum and is highly expressed in pancreatic beta cells and neuronal cells (34). The functional loss of wolframin causes the aggregation of misfolded protein in the endoplasmic reticulum, resulting in retinal ganglion cell death. Diabetes tends to appear as the first sign of Wolfram syndrome, followed by optic atrophy in the early twenties (35). Optic atrophy presented in about 84.4% of patients with Wolfram syndrome and the decline in visual acuity progressed to 20/200 or worse within 8 years from the onset of disease (36). However, P17 presented with optic atrophy as the first symptom in early childhood and was diagnosed with diabetes 2 years later. Her younger brother also had the same history of being diagnosed with optic atrophy first and then later with diabetes. Therefore, if isolated optic atrophy is diagnosed in early childhood and a genetic cause is strongly suspected, genetic evaluation of the patient is strongly recommended and regular check-up is necessary.

This study had several limitations. First, the study was limited by its retrospective design and all the patients in this study were of Korean ethnicity. Thus, it should be noted that other genetic backgrounds may have different profiles of gene variants. Although our targeted panel included 595 genes and deep intronic c.713-1075C>G in the *WFS1* gene, other deep intronic variants (Supplementary Table 7), secondary mitochondrial DNA variations or copy number variations could have been missed. Second, our panel also did not include the *PTPN23* gene, which was recently reported to be involved in hereditary optic atrophy (37). However, our targeted panel captures the poor coverage region efficiently in the *NR2F1* exon 1 (Supplementary Figure 7). Third, our panel did not include *DNAJC30* gene that is recently known as a cause of autosomal recessive LHON (38). We conducted exome re-analysis in 17 patients, but no candidate variants were found in *DNAJC30* gene. In addition, LHON sequencing was not performed in patients with congenital or infantile-onset optic atrophy. As childhood onset LHON had been reported (39, 40), this could be one of limitations of the study. Fourth, segregation analyses could not be performed for all the patients. Fifth, we did not perform functional studies to corroborate the pathogenicity of the novel variants identified in this study. Also, the minor allele frequency of c.305A>G *OPA1* variant was relatively high (5/248904), and unaffected mother of the proband also had this variant. Although previous studies reported this variant as disease-causing and non-penetrance was well characterized in OPA1-dominant optic atrophy (41, 42), this variant might be questionable in pathogenicity. Lastly, although we attempted to carefully exclude non-genetic causes of optic neuropathies, there may have been other non-genetic causes of optic atrophy that were missed in the unsolved patients.

This study demonstrated that NGS can be used for the diagnosis of patients with hereditary optic atrophy, with 31.6% of patients in our cohort having a definite diagnosis. This study emphasized that examining genotype-phenotype correlations and family segregation analyses are important in interpreting genetic variations in patients with hereditary optic atrophy. Accurate molecular diagnosis will enable ophthalmologists to conduct genetic counseling for future family planning and patient-specific diagnostic workups, including diabetes screening, auditory function tests, and cardiac evaluation. Given that optic atrophy has various causes, the careful collection of patient history, recognition of syndromic features, appropriate brain imaging, and laboratory investigations should be considered first to avoid unnecessary genetic investigations (Supplementary Figure 8).

## Data availability statement

The datasets for this article are not publicly available due to concerns regarding participant/patient anonymity. Requests to access the datasets should be directed to the corresponding author. Requests to access the datasets should be directed to JH, jinuhan@yuhs.ac.

## Ethics statement

This study was approved by the institutional review board of Gangnam Severance Hospital, Seoul, South Korea (3-2020-0063) and adhered to the tenets of the Declaration of Helsinki. Written informed consent to participate in this study was provided by the participants' legal guardian/next of kin.

## Author contributions

JH and S-HH contributed to conception and design of the study. YS and TK organized the database. YS and JH performed the statistical analysis and wrote the first draft of the manuscript. DW, SS, JC, S-TL, HL, and S-HH wrote sections of the manuscript. BL acquired and analyzed the data. All authors contributed to manuscript revision, read, and approved the submitted version.

## Funding

This study was supported by a Faculty Research Grant of Yonsei University College of Medicine (6-2017-0044) (6-2020-0146) and National Research Foundation of Korea (NRF) grant funded by the Korean Government (MSIT) (No. 2020R1C1C1007965).

## Conflict of interest

JC and S-TL were employed by Dxome Co., Ltd. The remaining authors declare that the research was conducted in the absence of any commercial or financial relationships that could be construed as a potential conflict of interest.

## Publisher's note

All claims expressed in this article are solely those of the authors and do not necessarily represent those of their affiliated organizations, or those of the publisher, the editors and the reviewers. Any product that may be evaluated in this article, or claim that may be made by its manufacturer, is not guaranteed or endorsed by the publisher.

## Abbreviations

NGS: next-generation sequencing
ES: exome sequencing
GS: genome sequencing
BBSOAS: Bosch-Boonstra-Schaff optic atrophy syndrome.
