# Supplementary material for: Genetic spectrum and characteristics of autosomal optic neuropathy in Korean: Use of next-generation sequencing in suspected hereditary optic atrophy
Source: Front Neurol. 2022 Aug 22;13:978532. doi: 10.3389/fneur.2022.978532 (PMC9441910; doi:10.3389/fneur.2022.978532)
Supplement: Supplementary file 1 [file Data_Sheet_1.docx]

Supplementary Table

# Supplementary Methods. Bioinformatic analysis of NGS, Annotation, Interpretation of Variants, Phenotype Review, and Consensus.

# Supplementary Table 1. Target panel genes associated with inherited eye diseases.

# Supplementary Table 2. Non-coding deep intronic or regulatory variants covered by the targeted panel.

# Supplementary Table 3. Optic Atrophy Genes in Online Mendelian Inheritance in Man.

# Supplementary Table 4. The clinical features of 39 patients with unsolved cases.

# Supplementary Table 5. Predictive pathogenicity scores and population frequency for 15 novel variants in this study (including online repository list)

# Supplementary Table 6. Possible genetic diagnosis in 1 unsolved patient.

# Supplementary Table 7. Non-coding deep intronic or regulatory variants not covered by the targeted panel.

# Supplementary Methods. Bioinformatic analysis of NGS, Annotation, Interpretation of Variants, Phenotype Review, and Consensus.

Library construction and next-generation sequencing of the proband’s DNA was conducted at the department of laboratory medicine, Yonsei University College of Medicine according to methods previously published.(1) Data analysis was performed primarily through our custom pipeline. Briefly, raw sequence data were mapped to GRCh37 (hg19) using the Burrows-Wheeler Aligner algorithm.(2) Then, we performed removal of duplicate reads, realignment of insertions and deletions, base quality recalibration, and variant calling using the Genome Analysis Toolkit.(3) Variants that were suspected to be pathogenic, likely pathogenic, or of unknown significance were confirmed by visual inspection of the bam file using Integrated Genomics Viewer version 2.8 (Broad Institute, Cambridge, MA, USA).

Split-read-based detection of large insertions and deletions was conducted using the Pindel(4) and Manta algorithms(5), and both of their results were crosschecked. Read-depth-based detection of copy number variations (CNVs) was conducted using the ExomeDepth software.(6) The CNVs detected by ExomeDepth were further cross-checked using our custom pipeline. This process retrieved base-level depth-of-coverage for each bam file using the SAMtools software and normalized the depths against those of other samples from the same batch. We performed off-target analysis of chromosomal CNVs using the CopywriteR software.(7) The presence of large exonic deletions and duplications was confirmed using the available MLPA kit (MRC Holland, Amsterdam, the Netherland). The chromosomal CNVs detected by off-target analysis were validated by Affymetrix Cytoscan 750K array (Genome build, hg19). The Affymetric Cytoscan 750K array contains 750,000 markers for copy number analysis, including 200,000 single nucleotide polymorphisms (SNPs) and 550,000 polymorphic probes. Data analysis was performed using the Chromosomal Analysis Suite software version 1.2.2.

First (step 1), variants were filtered by their frequencies in population control databases, including Genome Aggregation Database (non-TCGA dataset; frequencies were calculated according to ethnic subgroups), ESP6500, 1000 Genomes Project, and Korean Reference Genome Database. To filter common variant, we filtered variants with a minor allele frequency greater than 1% in recessive gene and 0.5% in autosomal dominant gene. Secondly (step 2), literature and database searches for previous reports and functional studies were performed using the RetNet database (RetNet; https://sph.uth.edu/retnet/), the Alamut Visual software and Human Gene Mutation Database professional database.(8) Pathogenic or benign evidence was scored when predictions of all in silico algorithms agreed. The following algorithms were used for predicting pathogenicity (Sorting Intolerant from Tolerant (SIFT),(9) PolyPhen-2,(10) MutationTaster,(11) Mutation Assessor,(12) Combined Annotation Dependent Depletion (CADD),(13) PhyloPMam,(14) PhyloPVert,(14) Protein Variation Effect Analyzer (PROVEAN),(15) and Functional Analysis through Hidden Markov Models(16, 17)). Splice site analysis was performed using the MaxEntScan, NNSPLICE, Human Solice Finder, GeneSplicer, and SpliceFinder-like algorithms implemented in the Alamut Visual software (Interactive Biosoftware, Rouen, France).(18) The interpretation of variants was done according to the 5-tier classification system recommended by the American College of Medical Genetics and Genomics and the Association for Molecular Pathology using a step-by-step approach (Figure S1 in the Supplement).(19) The systematic approaches for variant classification are also described in eMethods (in the Supplement).

Finally, the last step involved genetic specialists or laboratory physicians presenting a preliminary report to the patient’s attending physicians or pediatric ophthalmologists, which listed all possible pathogenic variants, likely pathogenic variants, and variants of unknown significance (VUSs). When pathogenic or likely pathogenic variants were consistent with the patient’s phenotype based on in-depth review by ophthalmologists, final validation using other confirmatory assays and a parental study was planned if available. VUSs, especially missense variants, were prioritized according to population frequency, American College of Medical Genetics score, and the patient’s ocular phenotype. A parental study was scheduled to detect de novo occurrence for the candidate pathogenic or likely pathogenic variants, and VUSs if all trios were available.

# Supplementary Table 1. 595 target genes associated with inherited eye diseases

| **Targeted genes** |
| --- |
| *ABCA4, ABCB6, ABCC6, ABHD12, ACO2, ACP6, ADAM9, ADAMTS10, ADAMTS18, ADAMTSL4, ADGRV1, ADIPOR1, AFG3L2, AGBL1, AGBL5, AGK, AGPS, AHDC1, AHI1, AHR, AIPL1, AKR1E2, ALDH18A1, ALDH1A3, ALMS1, AMOT, ANKS6, ANTXR1, AP3B1, APTX, ARFGAP2, ARHGEF10L, ARHGEF18, ARL13B, ARL2BP, ARL3, ARL6, ARMC9, ARSG, ASB10, ASRGL1, ATAD3A, ATF6, ATOH7, ATP1A3, ATP6V1A, ATP8A2, ATXN7, AUH, B3GLCT, B9D1, B9D2, BBIP1, BBS1, BBS10, BBS12, BBS2, BBS4, BBS5, BBS7, BBS9, BCOR, BEST1, BFSP1, BFSP2, BLM, BLOC1S3, BLOC1S6, BMP4, BMP7, C10orf11, C10orf2, C12orf57, C12orf65, C19orf12, C1QTNF5, C21orf2, C2orf71, C5orf42, C8orf37, CA4, CABP4, CACNA1A, CACNA1F, CACNA2D4, CACNB4, CAPN15, CAPN5, CASK, CBS, CC2D2A, CCDC28B, CCT2, CDH23, CDH3, CDHR1, CEP104, CEP120, CEP164, CEP19, CEP290, CEP41, CEP78, CEP83, CERKL, CHD7, CHM, CHMP4B, CHN1, CHRDL1, CHST1, CHST6, CIB2, CISD2, CLCC1, CLK2, CLN3, CLN5, CLN6, CLN8, CLRN1, CLUAP1, CNGA1, CNGA3, CNGB1, CNGB3, CNNM4, CNTNAP2, COL11A1, COL11A2, COL17A1, COL18A1, COL25A1, COL2A1, COL4A1, COL8A2, COL9A1, COL9A2, COL9A3, COX7B, CRB1, CRX, CRYAA, CRYAB, CRYBA1, CRYBA4, CRYBB1, CRYBB2, CRYBB3, CRYGC, CRYGD, CRYGS, CSPP1, CTC1, CTDP1, CTNNA1, CTNNB1, CTNS, CWC27, CYP1B1, CYP27A1, CYP4V2, CYP51A1, CYTH3, DCAF6, DCDC1, DCDC2, DCN, DHCR7, DHDDS, DHX38, DLG4, DNAJC19, DNM1L, DRAM2, DTHD1, DTNBP1, EDN3, EDNRB, EFEMP1, ELOVL1, ELOVL4, ELP4, EMC1, EPHA2, ERCC2, ERCC3, ERCC5, ERCC6, ERCC8, ESPN, EXOSC2, EYA1, EYS, FAM126A, FAM161A, FAT1, FBN1, FDXR, FKRP, FKTN, FLVCR1, FOXC1, FOXD1, FOXD3, FOXE3, FOXL2, FOXO1, FRAS1, FREM1, FREM2, FRMD7, FSCN2, FTL, FXN, FYCO1, FZD4, GABRB2, GALE, GALK1, GALNS, GALT, GCNT2, GDF3, GDF6, GJA1, GJA3, GJA8, GJC2, GLA, GNAT1, GNAT2, GNB3, GNPAT, GNPTG, GPR143, GPR179, GRIN1, GRIN2B, GRK1, GRM6, GSN, GUCA1A, GUCA1B, GUCY2D, HARS, HCCS, HESX1, HGSNAT, HK1, HMCN1, HMGB3, HMX1, HOXA1, HOXB1, HPS1, HPS3, HPS4, HPS5, HPS6, HSF4, IDH3B, IFT140, IFT172, IFT81, IFT88, IMPDH1, IMPG1, IMPG2, INPP5E, INVS, IQCB1, ITM2B, JAG1, JAM3, KCNA1, KCNJ13, KCNQ3, KCNV2, KCTD19, KIAA0556, KIAA0586, KIAA0753, KIAA1549, KIF11, KIF21A, KIF7, KIZ, KLHL7, KRT12, KRT3, LAMB2, LARGE1, LCA5, LCAT, LHX2, LIM2, LMX1B, LOXHD1, LRAT, LRIT3, LRP2, LRP5, LTBP2, LYST, LZTFL1, MAB21L2, MAF, MAFB, MAK, MAN2B1, MAPKAPK3, MC1R, MCOLN1, MERTK, MFN2, MFRP, MFSD6L, MFSD8, MIP, MITF, MKKS, MKS1, MLPH, MMACHC, MRE11A, MTO1, MTPAP, MTTP, MVK, MYF5, MYO7A, MYOC, NAA10, NBAS, NDP, NDUFS1, NECTIN3, NEK2, NEK8, NGLY1, NHS, NLRP3, NMNAT1, NPHP1, NPHP3, NPHP4, NR2E3, NR2F1, NRL, NTF4, NYX, OAT, OCA2, OCRL, OFD1, OPA1, OPA3, OPN1LW, OPN1MW, OPN1SW, OPTN, OTX2, OVOL2, P3H2, PANK2, PAX2, PAX3, PAX6, PCDH15, PCYT1A, PDE6A, PDE6B, PDE6C, PDE6D, PDE6G, PDE6H, PDHA1, PDZD7, PEX1, PEX10, PEX11B, PEX12, PEX13, PEX14, PEX16, PEX19, PEX2, PEX26, PEX3, PEX5, PEX5L, PEX6, PEX7, PGAP1, PHOX2A, PHYH, PIKFYVE, PITPNM3, PITX2, PITX3, PLA2G5, PLA2G6, PNPLA6, POC1B, POC5, POLG, POMGNT1, POMT1, POMT2, PPT1, PQBP1, PRCD, PRDM13, PRDM5, PRKAA2, PRKCG, PROM1, PRPF3, PRPF31, PRPF4, PRPF6, PRPF8, PRPH2, PRPS1, PRSS56, PXDN, PYGM, RAB18, RAB27A, RAB28, RAB3GAP1, RAB3GAP2, RARB, RAX, RAX2, RBP3, RBP4, RCBTB1, RD3, RDH11, RDH12, RDH5, RECQL4, REEP6, RERE, RGR, RGS9, RGS9BP, RHO, RIMS1, RLBP1, RNLS, ROBO3, ROM1, RP1, RP1L1, RP2, RP9, RPE65, RPGR, RPGRIP1, RPGRIP1L, RRM2B, RS1, RTN4IP1, SAG, SALL4, SAMD11, SC5D, SCAPER, SCLT1, SDCCAG8, SEC23A, SEMA4A, SETX, SH3PXD2B, SHH, SIL1, SIX3, SIX5, SIX6, SLC16A12, SLC1A1, SLC1A3, SLC24A1, SLC24A5, SLC25A16, SLC25A46, SLC2A1, SLC33A1, SLC35A2, SLC35A4, SLC38A8, SLC45A2, SLC4A11, SLC52A2, SLC7A14, SMCHD1, SNAI2, SNRNP200, SNX10, SNX3, SOX10, SOX2, SOX3, SOX5, SPATA7, SPG7, SRD5A3, SREBF2, SSBP1, STRA6, TACSTD2, TAT, TBK1, TCF4, TCTN1, TCTN2, TCTN3, TDRD7, TEAD1, TEK, TENM3, TFAP2A, TGFBI, TIMM8A, TIMP3, TK2, TMEM107, TMEM114, TMEM126A, TMEM138, TMEM216, TMEM231, TMEM237, TMEM67, TMEM70, TMEM98, TOPORS, TP63, TPP1, TRAF3IP1, TREX1, TRIM32, TRNT1, TRPM1, TSFM, TSPAN12, TTC21B, TTC8, TTLL5, TTPA, TUB, TUBB3, TUBGCP6, TULP1, TYMP, TYR, TYRP1, UBIAD1, UCHL1, UFSP2, UHMK1, UNC119, USH1C, USH1G, USH2A, USP45, VAX1, VCAN, VIM, VPS13B, VSX1, VSX2, WDPCP, WDR19, WDR34, WDR36, WFS1, WHRN, WRN, WT1, YME1L1, ZEB1, ZFP30, ZIC2, ZNF408, ZNF423, ZNF469, ZNF513, ZNHIT3* |

**Table S1**: Genes included in inherited eye diseases target enrichment. Listed are the genes included in the custom designed target enrichment along with the disease or phenotype associated with the gene according to Online Mendelian Inheritance in Man (OMIM), OMIM phenotype identification number, and OMIM or Gene Cards gene identification number. Genes are named according to HUGO Gene Nomenclature Committee (HUGO, <http://www.genenames.org/)> approved nomenclature. Bold and underlined genes were reported as causative gene of optic neuropathy or optic atrophy. Previously reported genes associated with optic atrophy (*ALG3,* *PDXK*, and *TBC1D20*) were absent in the targeted panel.

# Supplementary Table 2. Non-coding deep intronic or regulatory variants covered by the panel

| **Gene HGNC** | **Genomic location hg19** | **HGVS** | **Refseq** |
| --- | --- | --- | --- |
| *ABCA4* | Chr1:94526934 | c.1938-619A>G | NM_000350.2 |
| *ABCA4* | Chr1:94525509 | c.2160+584A>G | NM_000350.2 |
| *ABCA4* | Chr1:94576926 | c.302+68C>T | NM_000350.2 |
| *ABCA4* | Chr1:94509799 | c.3050+370C>T | NM_000350.2 |
| *ABCA4* | Chr1:94493272 | c.4539+1729G>T | NM_000350.2 |
| *ABCA4* | Chr1:94484082 | c.5196+1056A>G | NM_000350.2 |
| *ABCA4* | Chr1:94484001 | c.5196+1137G>A | NM_000350.2 |
| *ABCA4* | Chr1:94484001 | c.5196+1137G>T | NM_000350.2 |
| *ABCA4* | Chr1:94566773 | c.570+1798A>G | NM_000350.2 |
| *ABCA4* | Chr1:94468019 | c.6148-471C>T | NM_000350.2 |
| *ATP1A3* | Chr19: 42470891 | c.*196_*198dupCTC | NM_001256213.1 |
| *BBS1* | Chr11:66291105 | c.951+58C>T | NM_024649.4 |
| *BBS4* | Chr15:73001821 | c.77-220delA | NM_033028.4 |
| *BBS5* | Chr2:170354110 | c.619-27T>G | NM_152384.2 |
| *C5orf42* | Chr5: 37157484 | c.7957+288G>A | NM_023073.3 |
| *CEP290* | Chr12:88494960 | c.2991+1655A>G | NM_025114.3 |
| *CEP290* | Chr12:88462434 | c.6012-12T>A | NM_025114.3 |
| *CHM* | ChrX:85223644 | c.315-4587T>A | NM_000390.2 |
| *COL11A1* | Chr1:103488576 | c.1027-24A>G | NM_080629.2 |
| *COL11A1* | Chr1:103386637 | c.3744+437T>G | NM_080629.2 |
| *COL11A1* | Chr1:103491958 | c.781-450T>G | NM_080629.2 |
| *COL2A1* | Chr12:48379984 | c.1527+135G>A | NM_001844.4 |
| *CTNS* | Chr17: 3552117 | c.141-24T>C | NM_001031681.2 |
| *DHDDS* | Chr1:26774026 | c.441-24A>G | NM_024887.3 |
| *ELP4* | Chr11:31685945 | c.1143+14176C>A | NM_019040.4 |
| *ERCC6* | Chr10: 50681659 | c.2599-26A>G | NM_000124.3 |
| *ERCC8* | Chr5: 60223645 | c.173+1046A>G | NM_001290285.1 |
| *FKTN* | Chr9: 108368857 | c.648-1243G>T | NM_001079802.1 |
| *FOXC1* | Chr6:1610252 | c.-429C>G | NM_001453.2 |
| *FOXC1* | Chr6:1610437 | c.-244C>T | NM_001453.2 |
| *FOCX1* | Chr6:1613076 | c.*734A>T | NM_001453.2 |
| *FRMD7* | ChrX:131228285 | c.285-118C>T | NM_194277.2 |
| *FTL* | Chr19: 49468583 | c.[-178T>G;-182C>T] | NM_000146.3 |
| *FTL* | Chr19: 49468587 | c.-168G>A | NM_000146.3 |
| *FTL* | Chr19: 49468601 | c.-164C>A | NM_000146.3 |
| *FTL* | Chr19: 49468604 | c.-161C>T | NM_000146.3 |
| *FTL* | Chr19: 49468605 | c.-160A>G | NM_000146.3 |
| *FTL* | Chr19: 49468606 | c.-159G>C | NM_000146.3 |
| *FTL* | Chr19: 49468616 | c.-149G>C | NM_000146.3 |
| *GALT* | Chr9: 34646583 | c.-67-52_-67-49del | NM_000155.3 |
| *GALT* | Chr9: 34648519 | c.687+66T>A | NM_000155.3 |
| *GALT* | Chr9: 34649617 | c.1059+56C>T | NM_000155.3 |
| *GLA* | ChrX: 100654735 | c.639+919G>A | NM_000169.2 |
| *GLA* | ChrX: 100654793 | c.640-859C>T | NM_000169.2 |
| *GNAT2* | Chr1:110151229 | c.461+24G>A | NM_005272.3 |
| *GPR143* | ChrX:9711844 | c.659-131T>G | NM_000273.2 |
| *GPR143* | ChrX:9708630 | c.885+748G>A | NM_000273.2 |
| *IMPDH1* | Chr7: 128043703 | c.402+57G>A | NM_000883.3 |
| *LCAT* | Chr16: 67976512 | c.524-22T>C | NM_000229.1 |
| *MYO7A* | Chr11:76893448 | c.3109-21G>A | NM_000260.3 |
| *NF2* | Chr22: 30050946 | c.516+232G>A | NM_000268.3 |
| *NMNAT1* | Chr1:10003561 | c.-69C>T | NM_022787.3 |
| *NMNAT1* | Chr1:10003560 | c.-70A>T | NM_022787.3 |
| *OAT* | Chr10: 126100239 | c.199+303C>G | NM_000274.3 |
| *OCRL* | ChrX:128687279 | c.239-4023A>G | NM_000276.3 |
| *OFD1* | ChrX:13773245 | c.1130-22_1130-19delAATT | NM_003611.2 |
| *OFD1* | ChrX:13768358 | c.935+706A>G | NM_003611.2 |
| *OPN1MW* | ChrX: 153448055 | c.-112A>C | NM_000513.2 |
| *OVOL2* | Chr20:18038552 | c.-274T>G | NM_021220.2 |
| *OVOL2* | Chr20:18038585 | c.-307T>C | NM_021220.2 |
| *OVOL2* | Chr20:18038648 | c.-370T>C | NM_021220.2 |
| *PAX6* | Chr11:31816377 | c.524-41T>G | NM_000280.4 |
| *PDHA1* | ChrX: 19372579 | c.511-30G>A | NM_000284.3 |
| *PDHA1* | ChrX: 19373648 | c.759+26G>A | NM_000284.3 |
| *PDHA1* | ChrX: 19377850 | c.*79_*90dupAGTCAATGAAAT | NM_000284.3 |
| *PEX7* | Chr6: 137143759 | c.-45C>T | NM_000288.3 |
| *PITX2* | Chr4:111538827 | c.*454C>T | NM_000325.5 |
| *PPT1* | Chr1: 40539204 | c.*526_*529delATCA | NM_000310.3 |
| *PROM1* | Chr4: 15985995 | c.2281-26_2281-17del | NM_006017.2 |
| *PROM1* | Chr4:15989860 | c.2077-521A>G | NM_006017.2 |
| *PRPF31* | Chr19:54633399 | c.1374+654C>G | NM_015629.3 |
| *SDCCAG8* | Chr1: 243468435 | c.740+356C>T | NM_001350246.1 |
| *TIMM8A* | ChrX:100601671 | c.133-23A>C | NM_004085.3 |
| *TRNT1* | Chr3: 3188088 | c.609-26T>C | NM_001302946.1 |
| *USH2A* | Chr1:216247476 | c.5573-834A>G | NM_206933.2 |
| *USH2A* | Chr1:216064540 | c.7595-2144A>G | NM_206933.2 |
| *USH2A* | Chr1:216039721 | c.8845+628C>T | NM_206933.2 |
| *USH2A* | Chr1:215967783 | c.9959-4159A>G | NM_206933.2 |
| *WFS1* | Chr4: 6295693 | c.713-1075C>G | NM_001145853.1 |

# Supplementary Table 3. Optic Atrophy Genes in Online Mendelian Inheritance in Man

| **Locus** | **Gene HGNC** | **Location** | **Phenotype** | **Inheritance** |
| --- | --- | --- | --- | --- |
| OPA1 | *OPA1* | 3q29 | Optic atrophy 1 | AD |
| OPA2 | - | Xp11.4-p11.21 | Optic atrophy 2, X-linked | XL |
| OPA3 | *OPA3* | 19q13.32 | 3-methylglutaconic aciduria, type III | AR |
|  |  |  | Optic atrophy 3 with cataract | AD |
| OPA4 | *-* | 18q12.2-q12.3 | Optic atrophy 4 | - |
| OPA5 | *DNM1L* | 12p11.21 | Encephalopathy, lethal, due to defective mitochondrial peroxisomal fission 1 | AD, AR |
|  |  |  | Optic atrophy 5 | AD |
| OPA6 | *-* | 8q21-q22 | Optic atrophy 6 | AR |
| OPA7 | *TMEM126A* | 11q14.1 | Optic atrophy 7 | AR |
| OPA8 | *-* | 16q21-q22 | Optic atrophy 8 | AD |
| OPA9 | *ACO2* | 22q13.2 | ?Optic atrophy 9 | AR |
|  |  |  | Infantile cerebellar-retinal degeneration | AR |
| OPA10 | *RTN4IP1* | 6q21 | Optic atrophy 10 with or without ataxia, mental retardation, and seizures | AR |
| OPA11 | *YME1L1* | 10p12.1 | ?Optic atrophy 11 | AR |
| OPA12 | *AFG3L2* | 18p11.21 | Optic atrophy 12 | AD |
|  |  |  | Spastic ataxia 5, autosomal recessive | AR |
|  |  |  | Spinocerebellar ataxia 28 | AD |
| OPA13 | *SSBP1* | 7q34 | Optic atrophy with retinal and foveal abnormalities | AD |

# Supplementary Table 4. The clinical features of 39 unsolved cases

| **Pt** | **Initial diagnosis** | **Sequencing method** | **Final diagnosis** | **Sex** | **Age (y)** | **Onset age** | **Nystagmus** | **Refraction** | | **BCVA (OD/OS) logMAR** | **Fundus** | **Average RNFL thickness ^a^ (temporal, ㎛)** | | **ERG** | **Additional phenotypes** |
| --- | --- | --- | --- | --- | --- | --- | --- | --- | --- | --- | --- | --- | --- | --- | --- |
|  |  |  |  |  |  |  |  | **OD** | **OS** |  |  | **OD** | **OS** |  |  |
| 19 | BBSOAS | Targeted panel | unsolved | M | 15.9 | Infantile onset | Manifest latent nystagmus | -7 | -6 | 0.30/1.0 | Generalized optic atrophy | 50 | NA | NA | Delayed development |
| 20 | DOA | Targeted panel | unsolved | M | 8.7 | Early childhood onset | None | 0 | -0.25 | 0.30/0.15 | Temporal optic atrophy | 86 (47) | 86 (44) | NA | None |
| 21 | DOA | Targeted panel | unsolved | F | 8.2 | Early childhood onset | None | 2.25 | 2.25 | 0.30/0.30 | Generalized optic atrophy | 87 (68) | 88 (85) | NA | None |
| 22 | Unknown cause | Targeted panel | unsolved | F | 47.8 | Late onset | None | -0.5 | 0.5 | 2.8/0.5 | Generalized optic atrophy | 43 | 49 | NA | None |
| 23 | DOA | Targeted panel | unsolved | M | 4.8 | Early childhood onset | None | 0.13 | 0 | 0.3/0.3 | Temporal optic atrophy | 81 (70) | 76 (55) | NA | None |
| 24 | Unknown cause | Targeted panel | unsolved | F | 44.2 | Early childhood onset | 2Hz right beating jerk | -5.75 | -8 | 1.0/1.0 | Generalized optic atrophy | NA | NA | NA | None |
| 25 | DOA | Targeted panel | unsolved | M | 23.3 | Unknown | None | -7.25 | -7 | 0.05/0.10 | Temporal optic atrophy | 73 (61) | 72 (52) | Normal | None |
| 26 | DOA | Targeted panel | unsolved | M | 5.3 | Early childhood onset | Latent nystagmus | 1.25 | 1.5 | 0.30/0.70 | Temporal optic atrophy | NA | NA | NA | Delayed development, Cerebellar hypoplasia |
| 27 | Unknown cause | ES | unsolved | F | 42.2 | Infantile onset | 3Hz multidirectional nystagmus (mainly downbeat) | 1.5 | 1.25 | 0.52/0.70 | Generalized optic atrophy | NA | NA | Normal | None |
| 28 | Unknown cause | Targeted panel | unsolved | M | 61.9 | Early childhood onset | None | -0.5 | -0.5 | 0.30/0.05 | Temporal optic atrophy | 82 (26) | 82 (28) | NA | None |
| 29 | BBSOAS | Targeted panel | unsolved | M | 24.4 | Infantile onset | Latent nystagmus | -5.5 | -6 | 0.30/0.30 | Generalized optic atrophy, foveal hypoplasia | NA | 58 | NA | Facial dysmorphism |
| 30 | DOA | Targeted panel | unsolved | M | 29.3 | Infantile onset | None | -3.75 | -3.75 | 0.52/0.15 | Temporal optic atrophy, pseudodrusen | 83 (63) | 85 (59) | Decreased 30Hz photopic flicker response | Morton’s neuroma, talocalcaneal coalition, Raynaud’s syndrome |
| 31 | Unknown cause | Targeted panel | unsolved | M | 42.2 | Late onset | None | -1.25 | -1 | 0.0/0.0 | Temporal optic atrophy | 74 (47) | 80 (45) | NA | None |
| 32 | Unknown cause | Targeted panel | unsolved | F | 59.9 | Unknown | None | 0.5 | 0.25 | 0.15/0.30 | Temporal optic atrophy | 88 (37) | 89 (30) | NA | DM, Cerebral aneurysm |
| 33 | Unknown cause | Targeted panel | unsolved | M | 48.8 | Unknown | None | -7.13 | -7.5 | 0.22/0.05 | Generalized optic atrophy | 35 | 47 | NA | None |
| 34 | Unknown cause | Targeted panel | unsolved | M | 58.3 | Late onset | None | 0.25 | 0 | 0.15/0.10 | Temporal optic atrophy, drusen  Temporal optic atrophy | 98 (48) | 96 (46) | NA | None |
| 35 | BBSOAS | ES | unsolved | F | 23.5 | Unknown | Latent nystagmus | -10 | -9.88 | 0.15/0.10 | Generalized optic atrophy | NA | 46 | NA | None |
| 36 | BBSOAS | ES | unsolved | F | 30.1 | Unknown | Latent nystagmus | 4.5 | 4.5 | 0.0/0.0 | Generalized optic atrophy | NA | NA | NA | Epilepsy, Delayed development |
| 37 | Unknown cause | ES | unsolved | M | 48.5 | Late onset | None | -5.5 | -5.5 | 0.7/0.0 | Generalized optic atrophy | 59 | 45 | Normal | High uric acid level |
| 38 | DOA | Targeted panel | unsolved | F | 7.6 | Early childhood onset | None | 1.25 | 1.13 | 1.0/1.0 | Generalized optic atrophy | 89 | 74 | NA | None |
| 39 | Unknown cause | Targeted panel | unsolved | M | 42.5 | Late onset | None | -6 | -5.5 | 0.52/0.40 | Generalized optic atrophy | 79 | 74 | NA | None |
| 40 | BBSOAS | Targeted panel | unsolved | F | 4.9 | Infantile onset | Gaze-evoked nystagmus | 1.63 | 1.63 | 0.3/1.0 | Temporal optic atrophy | 79 (61) | 74 (56) | NA | Delayed development, Facial dysmorphism |
| 41 | Infantile cerebellar retinal degeneration | ES | unsolved | M | 3.8 | Infantile onset | Multidirectional nystagmus | -0.5 | -0.5 | 2.3/2.3 | Generalized optic atrophy, Diffuse granular retinal dystrophy | NA | NA | Flat response at photopic ERG | Delayed development, Hypotonia, Cerebellar atrophy |
| 42 | Unknown cause | ES | unsolved | M | 6.8 | Early childhood onset | None | NA | NA | 0.2/1 | Temporal optic atrophy | NA | NA | NA | None |
| 43 | Unknown cause | ES | unsolved | F | 27.8 | Late onset | None | NA | NA | 0.4.0.3 | Temporal optic atrophy | 57 (39) | 65 (33) | NA | None |
| 44 | Unknown cause | ES | unsolved | F | 49.1 | Late onset | None | NA | NA | 0.7/0.52 | Generalized optic atrophy | 57 | 58 | NA | None |
| 45 | Unknown cause | Targeted panel | unsolved | F | 8.8 | Early childhood onset | .None | 2 | 1.75 | 0.04/0.09 | Generalized optic atrophy | 76 | 54 | NA | None |
| 46 | Unknown cause | Targeted panel | unsolved | M | 9.9 | Early childhood onset | None | -4 | -4.25 | 0.22/0.15 | Generalized optic atrophy | 80 | 80 | NA | None |
| 47 | Unknown cause | Targeted panel | unsolved | F | 45.9 | Late onset | None | -4.5 | -2.25 | 0/0 | Temporal optic atrophy | 97 (73) | 73 (34) | NA | None |
| 48 | Unknown cause | Targeted panel | unsolved | M | 18.8 | Early childhood onset | None | -3 | -4.75 | 0.09/1.22 | Generalized optic atrophy | 57 | 51 | NA | None |
| 49 | Unknown cause | Targeted panel | unsolved | F | 8.8 | Infantile onset | None | -2.75 | -3 | 0/0 | Generalized optic atrophy | 68 | 83 | NA | None |
| 50 | Unknown cause | Targeted panel | unsolved | M | 1.8 | Infantile onset | Intermittent 2Hz left beat jerk nystagmus | 0 | 0.25 | NA/NA | Generalized optic atrophy | NA | NA | Flat response at photopic ERG | None |
| 51 | Unknown cause | Targeted panel | unsolved | F | 21.6 | Late onset | None | -0.5 | -0.25 | 0.52/0.52 | Generalized optic atrophy | 65 | 44 | NA | None |
| 52 | Unknown cause | Targeted panel | unsolved | F | 43.8 | Late onset | None | -2 | -1.75 | 0/0 | Temporal optic atrophy | 55 (34) | 55 (26) | NA | None |
| 53 | Unknown cause | ES | unsolved | F | 7.9 | Early childhood onset | None | -0.25 | 0.25 | 0.15/0.15 | Generalized optic atrophy | 80 | 79 | NA | None |
| 54 | Unknown cause | Targeted panel | unsolved | M | 56.5 | Late onset | None | 0.5 | 0.75 | 0/0 | Temporal optic atrophy | 82 (32) | 74 (26) |  | None |
| 55 | Unknown cause | Targeted panel | unsolved | F | 5.7 | Early childhood onset | None | -8.25 | -8.25 | 0.09/0.09 | Generalized optic atrophy | 69 | 78 | NA | None |
| 56 | Unknown cause | ES | unsolved | M | 55.2 | Late onset | None | -0.5 | -1.25 | 0.39/2.3 | Generalized optic atrophy | 55 | 76 | NA | None |
| 57 | Unknown cause | ES | unsolved | M | 50.5 | Late onset | None | -7.25 | -3 | 0.39/0.22 | Temporal optic atrophy | 53 (35) | 47 (18) | NA | None |

BBSOAS = Bosch-Boonstra-Schaff Optic Atrophy Syndrome; ES = Exome sequencing; GS = Genome sequencing; Targeted panel/ES = Targeted panel sequencing followed by ES; ES/GS = Exome sequencing followed by GS; BCVA = Best Corrected Visual Acuity; CSNB=Congenital Stationary Night Blindness; DM = Diabetes mellitus; DOA=Dominant Optic Atrophy; F = Female; M = Male; NA = Not Available; UCSM = no constant, no steady, no maintained fixation; HC: Head circumference ;OD=right; OS=left; SOPH=Short stature with Optic atrophy and Pelger-Huet anomaly; Infantile onset ≤ 1 year old; Early childhood onset < 13 year old; Late onset ≥ 13 years old ^a^In the case with temporal optic atrophy, temporal RNFL thickness was revealed in parenthesis below the average thickness.

# Supplementary Table 5. Predictive pathogenicity scores and population frequency for 18 causative variants in this study.

| **Patient number** | **P1** | **P2** | **P3** | **P4** | **P5** | **P6** | **P7** | **P8** | **P9** | **P10** | **P11** |
| --- | --- | --- | --- | --- | --- | --- | --- | --- | --- | --- | --- |
| **Gene** | *OPA1* | *OPA1* | *OPA1* | *OPA1* | *OPA1* | *OPA1* | *OPA1* | *NR2F1* | *NR2F1* | *NR2F1* | *NR2F1* |
| **Variant** | c.2297dup | c.1240A>C | c.795_798del | c.305A>G | c.1202G>A | c.784A>T | c.1620_1622del | c.513C>G | c.91_93dup | c.51_69dup | c.1080C>A |
| **Genomic position** | Chr3:g.193377292dup | Chr3:g.193361344A>C | Chr3:g.193354995_193354998del | Chr3:g.193332784A>G | Ch43:g: 193361223G>A | Chr3:g.193354984A>T | Chr3:g.193364884_193364886del | Chr5:g.92923672C>G | Chr5:g.92920820_92920822dupCGC | Chr5:g.92920780_92920798dup | Chr5(GRCh37):g.92929356C>A |
| **SIFT^1^** | NA | 0 | NA | 0 | 0 | NA | NA | NA | NA | NA | NA |
| **PolyPhen-2^2^** | NA | 1 | NA | 0.99 | 1 | NA | NA | NA | NA | NA | NA |
| **MutationAssesor^3^** | NA | NA | NA | NA | NA | NA | NA | NA | NA | NA | NA |
| **MutationTaster^4^** | **1.0** | NA | **1.0** | 0.99 | **0.99** | **1.0** | NA | **0.99** | **0.99** | **1.0** | **0.99** |
| **phyloMam^5^** | NA | 2.206 | NA | 2.285 | 2.732 | 2.02 | NA | 0.637 | NA | NA | 0.493 |
| **phyloVert^6^** | NA | 3.34 | NA | 4.3449 | 6.005 | 4.7 | NA | 1.243 | NA | NA | 2.183 |
| **CADD^7^** | **35** | **26.5** | **35** | **25.8** | **28.9** | **44** | **22.4** | **35** | **19.92** | **24** | **36** |
| **PROVEAN^8^** | NA | -5.63 | NA | -6.64 | -6.58 | NA | NA | NA | NA | NA | NA |
| **FATHMM^9^** | **0.991** | **0.959** | **0.991** | **0.775** | **0.926** | **0.991** | **0.997** | **0.985** | 0.140 | 0.470 | **0.931** |
| **GnomAD^10^** | Not found | Not found | Not found | 0 | Not found | Not found | Not found | Not found | Not found | Not found | Not found |
| **GnomAD_EA^11^** | Not found | Not found | Not found | 5/248904 | Not found | Not found | Not found | Not found | Not found | Not found | Not found |
| **ACMG^12^ Classification** | Pathogenic | Pathogenic | Pathogenic | Likely pathogenic | Likely pathogenic | Likely pathogenic | Likely pathogenic | Likely pathogenic | Likely pathogenic | Pathogenic | Pathogenic |
| **Pathogenicity**  **Criteria** | PVS1, PM2, PP3 | PS2,PM1,PM2,PP3,PP5 | PVS1, PM1, PM2, PM4, PP3, PP5 | PM1,PM2,PP3,PP4 | PM1, PM2,PP3,PP5 | PVS1, PM2 | PS3,PM2,PP1,PP3,PP4 | PVS1, PM2 | PM1, PM2, PP2, PP3 | PVS1, PM1, PM2 | PVS1, PM2 |
| **Online repository** | https://databases.lovd.nl/shared/individuals/00412266 |  | https://databases.lovd.nl/shared/variants/0000871047 |  |  | https://databases.lovd.nl/shared/variants/0000871048 |  | https://databases.lovd.nl/shared/variants/0000871049#00014783 | https://databases.lovd.nl/shared/variants/0000871050#00014783 | https://databases.lovd.nl/shared/variants/0000794207#00014783 | https://databases.lovd.nl/shared/variants/0000871051#00014783 |

Legend: Variant genomic positions are reported according to GRCh37.^1^Sorting Intolerant From Tolerant (SIFT) values ≤ 0.05 are predicted pathogenic; ^2^Polymorphism Phenotyping v2 HumVar (PolyPhen-2) values ≥ 0.09 are predicted pathogenic; ^3^Mutation Assessor (MA) values ≥ 1.9 are predicted pathogenic; ^4^Mutation Taster (MT) values ≥ 0.5 are predicted pathogenic; ^5^Phylogenetic P value mammals (PhyloPMam_avg) values > 2.3 are predicted pathogenic; ^6^Phylogenetic P value vertebrates (PhyloVert_avg) values > 4 are predicted pathogenic; ^7^Combined Annotation – Dependent Depletion (CADD phred) values ≥15 are predicted pathogenic; ^8^Protein Variation Effect Analyzer (PROVEAN) values ≤ -2.5 are predicted pathogenic; ^9^Functional Annotation through Hidden Markov Model (FATHMM)-v2.3 MKL and FATHMM-Indel values above 0.5 are predicted to be deleterious; ^10^Genome Aggregation Database (GnomAD); ^11^Genome Aggregation Database_EastAsian (EA); ^12^American College of Medical Genetics (ACMG); NA = not available.

(Continued)

| **Patient number** | **P12** | **P13** | **P14** | **P14** | **P15** | **P15** | **P16** | **P16** | **P17** | **P17** | **P18** |
| --- | --- | --- | --- | --- | --- | --- | --- | --- | --- | --- | --- |
| **Gene** | *SOX5* | *SPG7* | *NBAS* | *NBAS* | *PTPN23* | *PTPN23* | *TMEM126A* | *TMEM126A* | *WFS1* | *WFS1* | *SSBP1* |
| **Variant** | Whole gene deletion | c.1224T>G | c.3494del | c.5740C>T | c.3768del | c.4886C>G | c.28del | c.163C>T | c.631+1del | c.2262_2263del | c.364A>G |
| **Genomic position** |  | Chr16:g.89598944T>G | Chr2:g.15519822del | Chr2:g.15378795G>A | Chr3:g.47453061del | Chr3:g.47454650C>G | Chr11:g.85361327del | Chr11:g.85365183C>T | Chr4: g.6293095del | Chr4: g.6303784_6303785del | Chr7(GRCh37):g.141445345A>G |
| **SIFT^1^** | NA | **0** | NA | **0** | NA | **0** | NA | NA | NA | NA | 0.03 |
| **PolyPhen-2^2^** | NA | **1** | NA | **0.964** | NA | **0.997** | NA | NA | NA | NA | 0.86 |
| **MutationAssesor^3^** | NA | **2.825** | NA | **2.83** | NA | **2.57** | NA | NA | NA | NA | NA |
| **MutationTaster^4^** | NA | **0.99** | **1.0** | **1.0** | **1.0** | **0.99** | **0.99** | **0.99** | **1.0** | NA | NA |
| **phyloMam^5^** | NA | -0.83 | NA | 1.461 | NA | 2.221 | NA | 2.891 | NA | NA | 2.078 |
| **phyloVert^6^** | NA | -0.21 | NA | 1.436 | NA | **4.695** | NA | 1.829 | NA | NA | 4.443 |
| **CADD^7^** | NA | **22.2** | **34** | **24.9** | **31** | **25.5** | **16.74** | **38** | **22.7** | **35** | 24.7 |
| **PROVEAN^8^** | NA | **-3.7** | NA | **-4.93** | NA | **-4.29** | NA | NA | NA | NA | NA |
| **FATHMM^9^** | NA | **0.645** | **0.934** | **0.942** | **0.990** | **0.990** | 0.106 | 0.237 | **0.985** | NA | NA |
| **GnomAD^10^** | Not found | Not found | Not found | Not found | Not found | Not found | 3/249618 | Not found | Not found | Not found | Not found |
| **GnomAD_EA^11^** | Not found | Not found | Not found | Not found | Not found | Not found | 3/18358 | 8/251482 | Not found | Not found | Not found |
| **ACMG^12^ Classification** | Pathogenic | Likely pathogenic | Likely pathogenic | Likely pathogenic | Pathogenic | Uncertain Significance | Likely pathogenic | Likely pathogenic | Likely pathogenic | Likely pathogenic | Likely pathogenic |
| **Pathogenicity**  **Criteria** | PS2,PS3,PM1 | PS2, PM1, PM2 | PVS1, PM2 | PM2, PM5, PP3, PP4 | PVS1, PM2, PP3 | PM2, PM3, PP3 | PVS1, PM2 | PVS1,PM2 | PVS1, PM2 | PVS1, PM2 | PM1, PM2,  PP2, PP3 |
| **Online repository** |  | https://databases.lovd.nl/shared/variants/0000794125#00020169 | https://databases.lovd.nl/shared/variants/0000793916#00014309 | https://databases.lovd.nl/shared/variants/0000793915#00014309 | https://databases.lovd.nl/shared/variants/0000794691#00017154 | https://databases.lovd.nl/shared/variants/0000794692#00017154 | https://databases.lovd.nl/shared/variants/0000871052#00021362 |  | https://databases.lovd.nl/shared/variants/0000871053#00023835 |  | https://databases.lovd.nl/shared/variants/0000794693#00020333 |

Legend: Variant genomic positions are reported according to GRCh37.^1^Sorting Intolerant From Tolerant (SIFT)1 values ≤ 0.05 are predicted pathogenic; ^2^Polymorphism Phenotyping v2 HumVar (PolyPhen-2) values ≥ 0.09 are predicted pathogenic; ^3^Mutation Assessor (MA) values ≥ 1.9 are predicted pathogenic; ^4^Mutation Taster (MT) values ≥ 0.5 are predicted pathogenic; ^5^Phylogenetic P value mammals (PhyloPMam_avg) values > 2.3 are predicted pathogenic; ^6^Phylogenetic P value vertebrates (PhyloVert_avg) values > 4 are predicted pathogenic; ^7^Combined Annotation – Dependent Depletion (CADD phred) values ≥15 are predicted pathogenic; ^8^Protein Variation Effect Analyzer (PROVEAN) values ≤ -2.5 are predicted pathogenic; ^9^Functional Annotation through Hidden Markov Model (FATHMM)-v2.3 MKL and FATHMM-Indel; ^10^Genome Aggregation Database (GnomAD); ^11^Genome Aggregation Database_EastAsian (EA); ^12^American College of Medical Genetics (ACMG); NA = not available.

# Supplementary Table 6. Possible candidate causative variants in 1 unsolved patient

| **Patient No.** | **Gene** | **Variants** | **Zygosity** | **Segregation** | **gnomAD MAF** | ***SIFT*** | ***Poly-***  ***Phen2*** | ***CADD*** | **Exon**  **(Intron)** | **Literatures** | **Domain** | **ACMG** | **Accession ID**  **for transcript** |
| --- | --- | --- | --- | --- | --- | --- | --- | --- | --- | --- | --- | --- | --- |
| 41 | *ACO2* | c.250C>T:p.(Arg84*) | Hetero | NA | Not found | NA | NA | 35 | 3/18 | Novel | Acotinase domain | P | NM_001098.2 |

Legend : CADD hg19 v1.6 was used for scoring. gnomAD v2.1.1 was used for calculating the minor allele frequency of the variant. ACMG/AMP guideline: The American College of Medical Genetics and Genomics (ACMG) and the Association for Molecular Pathology (AMP) 2015 updated standards and guidelines for the clinical interpretation of sequence variants. CADD = Combined Annotation Dependent Depletion; gnomAD = genome aggregation database; LP = likely pathogenic; MAF = minor allele frequency; NA = not available; P = pathogenic

# Supplementary Table 7. Non-coding deep intronic or regulatory variants not covered by the panel

| **Gene HGNC** | **Genomic location hg19** | **HGVS** | **Refseq** |
| --- | --- | --- | --- |
| *OPA1* | Chr3(GRCh37):g.193374829G>C | c.2014-40G>C | NM_015560.2 |
| *SPG7* | Chr16(GRCh37):g.89577853A>G | c.286+853A>G | NM_003119.3 |
| *TIMM8A* | ChrX(GRCh37):g.100601671T>G | c.133-23A>C | NM_004085.3 |
| *WFS1* | Chr4(GRCh37):g.6271563_6271568dup | c.-184_-179dupTGCCCC | NM_006005.3 |
| *WFS1* | Chr4(GRCh37):g.6271704G>T | c.-43G>T | NM_006005.3 |
| *WFS1* | Chr4(GRCh37):g.6271741G>T | c.-6G>T | NM_006005.3 |

**References**

1. Rim JH, Lee ST, Gee HY, Lee BJ, Choi JR, Park HW, et al. Accuracy of Next-Generation Sequencing for Molecular Diagnosis in Patients with Infantile Nystagmus Syndrome. *JAMA Ophthalmol* (2017) 135(12):1376-85. Epub 2017/11/18. doi: 10.1001/jamaophthalmol.2017.4859.

2. Li H, Durbin R. Fast and Accurate Short Read Alignment with Burrows-Wheeler Transform. *Bioinformatics (Oxford, England)* (2009) 25(14):1754-60. Epub 2009/05/20. doi: 10.1093/bioinformatics/btp324.

3. McKenna A, Hanna M, Banks E, Sivachenko A, Cibulskis K, Kernytsky A, et al. The Genome Analysis Toolkit: A Mapreduce Framework for Analyzing Next-Generation DNA Sequencing Data. *Genome research* (2010) 20(9):1297-303. Epub 2010/07/21. doi: 10.1101/gr.107524.110.

4. Ye K, Schulz MH, Long Q, Apweiler R, Ning Z. Pindel: A Pattern Growth Approach to Detect Break Points of Large Deletions and Medium Sized Insertions from Paired-End Short Reads. *Bioinformatics (Oxford, England)* (2009) 25(21):2865-71. Epub 2009/06/30. doi: 10.1093/bioinformatics/btp394.

5. Chen X, Schulz-Trieglaff O, Shaw R, Barnes B, Schlesinger F, Kallberg M, et al. Manta: Rapid Detection of Structural Variants and Indels for Germline and Cancer Sequencing Applications. *Bioinformatics (Oxford, England)* (2016) 32(8):1220-2. Epub 2015/12/10. doi: 10.1093/bioinformatics/btv710.

6. Plagnol V, Curtis J, Epstein M, Mok KY, Stebbings E, Grigoriadou S, et al. A Robust Model for Read Count Data in Exome Sequencing Experiments and Implications for Copy Number Variant Calling. *Bioinformatics (Oxford, England)* (2012) 28(21):2747-54. Epub 2012/09/04. doi: 10.1093/bioinformatics/bts526.

7. Kuilman T, Velds A, Kemper K, Ranzani M, Bombardelli L, Hoogstraat M, et al. Copywriter: DNA Copy Number Detection from Off-Target Sequence Data. *Genome biology* (2015) 16:49. Epub 2015/04/19. doi: 10.1186/s13059-015-0617-1.

8. Stenson PD, Mort M, Ball EV, Evans K, Hayden M, Heywood S, et al. The Human Gene Mutation Database: Towards a Comprehensive Repository of Inherited Mutation Data for Medical Research, Genetic Diagnosis and Next-Generation Sequencing Studies. *Hum Genet* (2017) 136(6):665-77. Epub 2017/03/30. doi: 10.1007/s00439-017-1779-6.

9. Kumar P, Henikoff S, Ng PC. Predicting the Effects of Coding Non-Synonymous Variants on Protein Function Using the Sift Algorithm. *Nat Protoc* (2009) 4(7):1073-81. Epub 2009/06/30. doi: 10.1038/nprot.2009.86.

10. Adzhubei IA, Schmidt S, Peshkin L, Ramensky VE, Gerasimova A, Bork P, et al. A Method and Server for Predicting Damaging Missense Mutations. *Nat Methods* (2010) 7(4):248-9. Epub 2010/04/01. doi: 10.1038/nmeth0410-248.

11. Schwarz JM, Rödelsperger C, Schuelke M, Seelow D. Mutationtaster Evaluates Disease-Causing Potential of Sequence Alterations. *Nat Methods* (2010) 7(8):575-6. Epub 2010/08/03. doi: 10.1038/nmeth0810-575.

12. Reva B, Antipin Y, Sander C. Predicting the Functional Impact of Protein Mutations: Application to Cancer Genomics. *Nucleic Acids Res* (2011) 39(17):e118. Epub 2011/07/06. doi: 10.1093/nar/gkr407.

13. Rentzsch P, Witten D, Cooper GM, Shendure J, Kircher M. Cadd: Predicting the Deleteriousness of Variants Throughout the Human Genome. *Nucleic Acids Res* (2019) 47(D1):D886-d94. Epub 2018/10/30. doi: 10.1093/nar/gky1016.

14. Pollard KS, Hubisz MJ, Rosenbloom KR, Siepel A. Detection of Nonneutral Substitution Rates on Mammalian Phylogenies. *Genome Res* (2010) 20(1):110-21. Epub 2009/10/28. doi: 10.1101/gr.097857.109.

15. Choi Y, Sims GE, Murphy S, Miller JR, Chan AP. Predicting the Functional Effect of Amino Acid Substitutions and Indels. *PLoS One* (2012) 7(10):e46688. Epub 2012/10/12. doi: 10.1371/journal.pone.0046688.

16. Shihab HA, Rogers MF, Gough J, Mort M, Cooper DN, Day IN, et al. An Integrative Approach to Predicting the Functional Effects of Non-Coding and Coding Sequence Variation. *Bioinformatics* (2015) 31(10):1536-43. Epub 2015/01/15. doi: 10.1093/bioinformatics/btv009.

17. Ferlaino M, Rogers MF, Shihab HA, Mort M, Cooper DN, Gaunt TR, et al. An Integrative Approach to Predicting the Functional Effects of Small Indels in Non-Coding Regions of the Human Genome. *BMC Bioinformatics* (2017) 18(1):442. Epub 2017/10/08. doi: 10.1186/s12859-017-1862-y.

18. Xiong HY, Alipanahi B, Lee LJ, Bretschneider H, Merico D, Yuen RK, et al. Rna Splicing. The Human Splicing Code Reveals New Insights into the Genetic Determinants of Disease. *Science (New York, NY)* (2015) 347(6218):1254806. Epub 2014/12/20. doi: 10.1126/science.1254806.

19. Richards S, Aziz N, Bale S, Bick D, Das S, Gastier-Foster J, et al. Standards and Guidelines for the Interpretation of Sequence Variants: A Joint Consensus Recommendation of the American College of Medical Genetics and Genomics and the Association for Molecular Pathology. *Genet Med* (2015) 17(5):405-24. Epub 2015/03/06. doi: 10.1038/gim.2015.30.
