# Supplementary material for: Genetic spectrum and characteristics of autosomal optic neuropathy in Korean: Use of next-generation sequencing in suspected hereditary optic atrophy
Source: Front Neurol. 2022 Aug 22;13:978532. doi: 10.3389/fneur.2022.978532 (PMC9441910; doi:10.3389/fneur.2022.978532)
Supplement: Supplementary file 2 [file Data_Sheet_2.pdf]

# *Supplementary Figure*

1 **Supplementary Figure 1. Schematic diagram of next-generation sequencing analysis workflow.**

2 **Supplementary Figure 2. The distribution of age at referral for genetic testing.**

3 **Supplementary Figure 3. (P41) Possible diagnosis of infantile retinal cerebellar degeneration.**

4 **Supplementary Figure 4. Pedigree charts, fundus photographs, optical coherence tomographs and visual fields in 6 patients with *OPA1*-mutations.**

5 **Supplementary Figure 5. The novel c.631+1del variant in *WFS1* gene causes disruption of canonical splicing donor sites.**

6 **Supplementary Figure 6. (P10) Pedigree chart, Fundus photographs and optic coherence tomography in Bosch Boonstra Schaaf optic atrophy syndrome.**

7 **Supplementary Figure 7. The low coverage region of *NR2F1* exon 1 region (hg19 position- chr5:92,920,730~92,920,890) due to low-complex repetitive sequence.**

8 **Supplementary Figure 8. Schematic representation of the diagnostic workflow for suspected hereditary optic atrophy.**

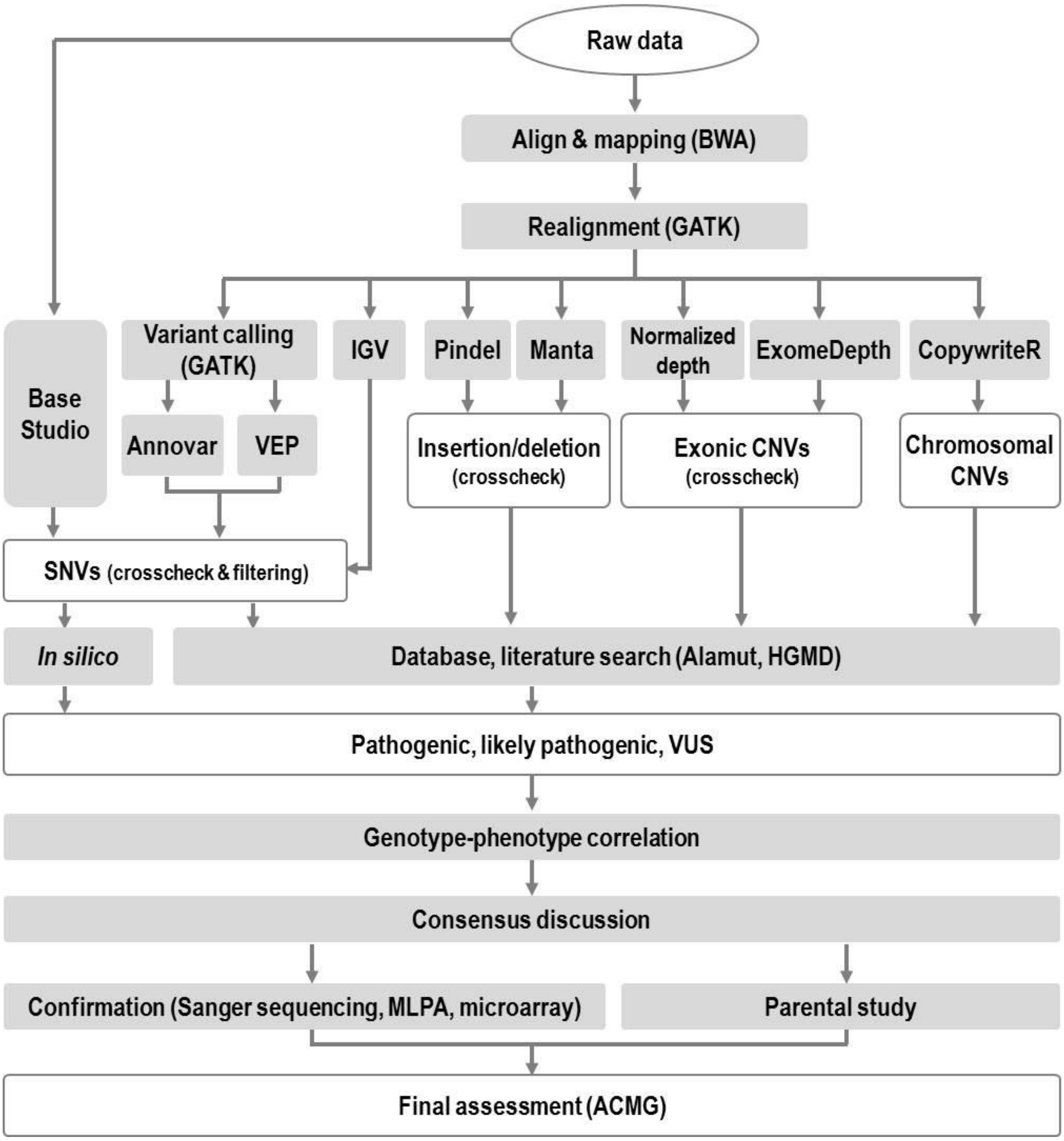


9 **Supplementary Figure 1. Schematic diagram of next-generation sequencing analysis work-flow**

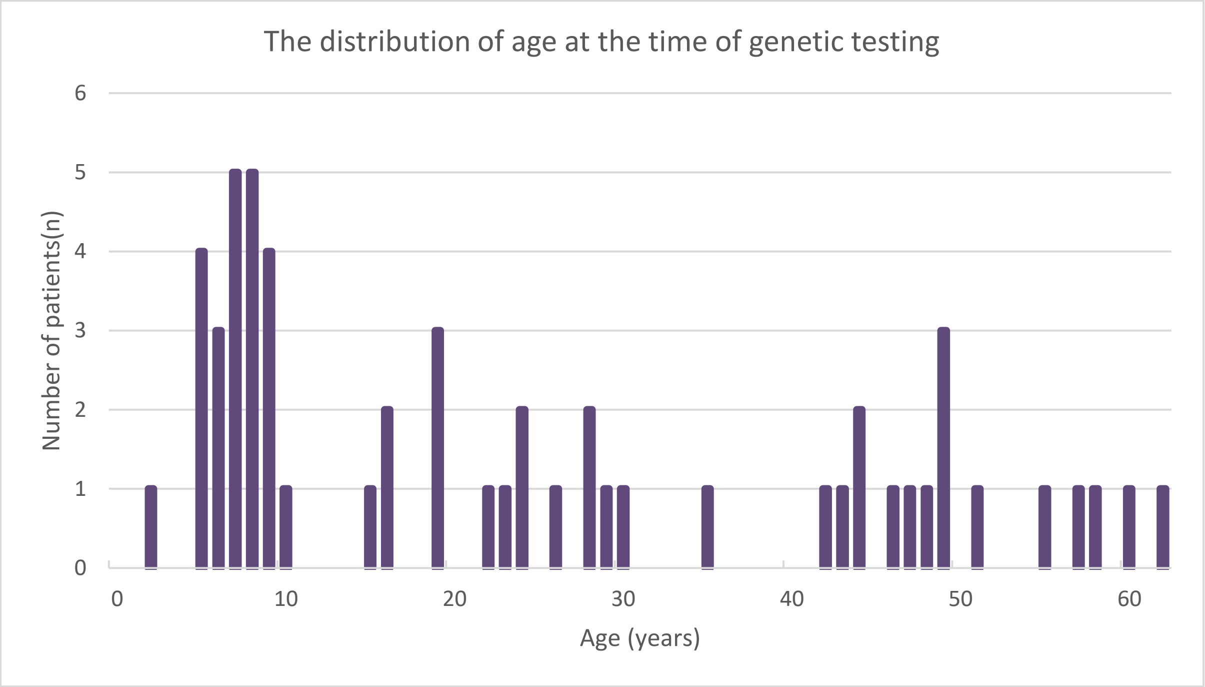


**Supplementary Figure 2. The distribution of age at referral for genetic testing.**

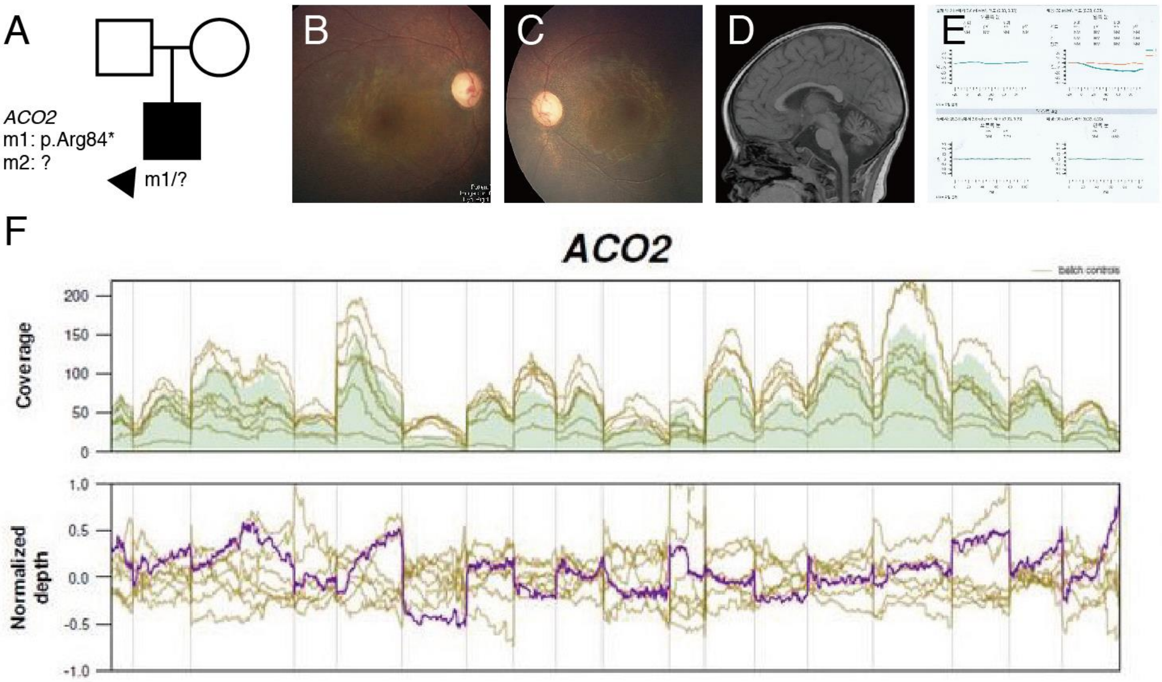


**Supplementary Figure 3. (P41)** Possible diagnosis of infantile retinal cerebellar degeneration. (A) Pedigree and next-generation sequencing analysis showing a heterozygous c.250C>T:p.(Arg83*) variant in *ACO2* gene. (B, C) Fundus photographs showing optic atrophy and granular pigmentary retinal dystrophy. (D) Sagittal brain magnetic resonance imaging showed cerebellar hypoplasia. (E) Dark-adapted ERG showed extinguished response in 0.01 and 3.0 dark adapted responses. (F) No copy number variation was detected in customized ExomDepth software.[6] The yellow lines indicated depth of coverage in same batch samples. The purple line showed normalized depth of coverage in P41. We could not identify a second hidden genetic variation in *ACO2* gene.

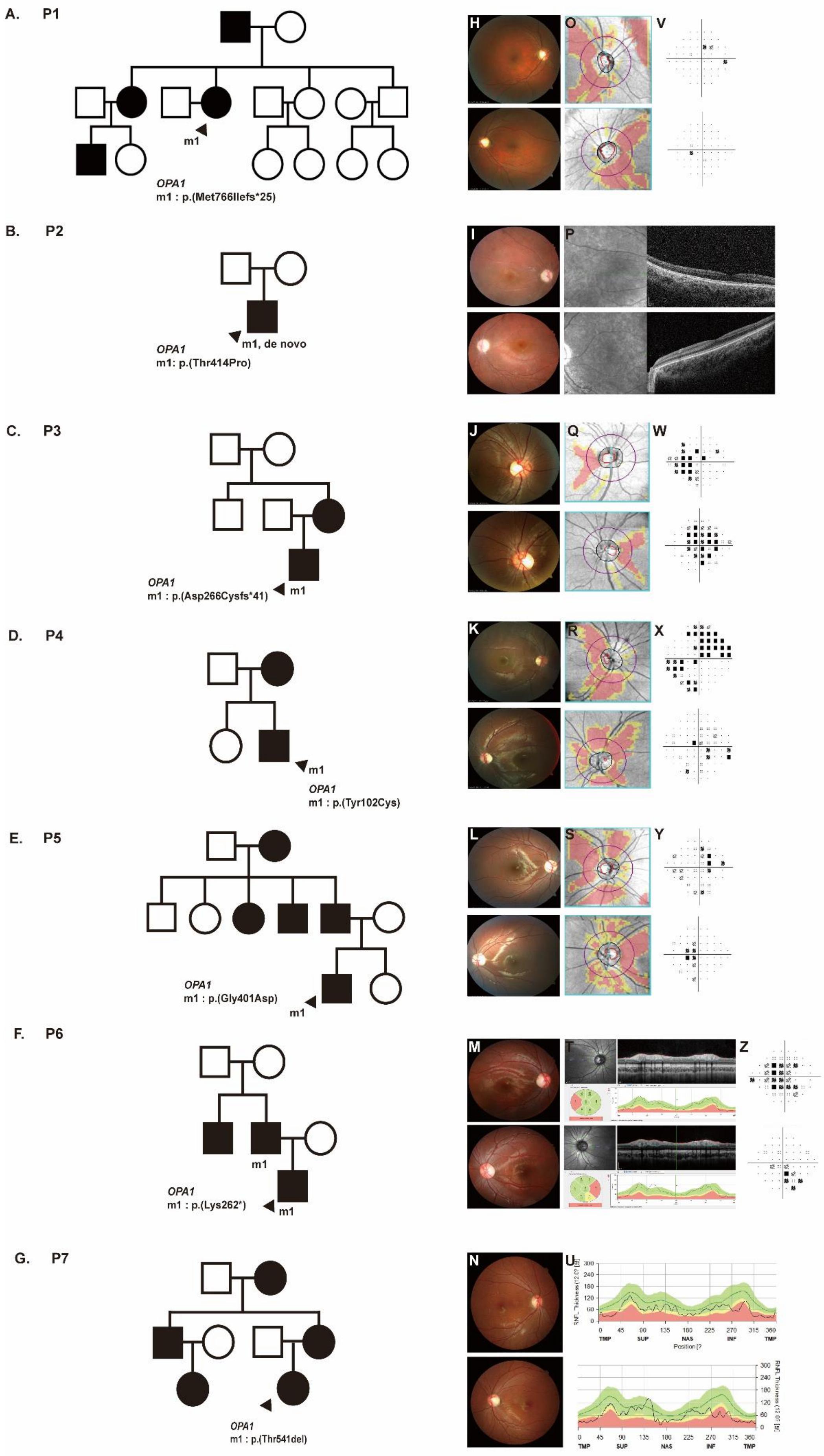
A. P1
OPA1
m1 : p.(Met766Ilefs*25)
B. P2
m1, de novo
OPA1
m1: p.(Thr414Pro)
C. P3
OPA1
m1 : p.(Asp266Cysfs*41)
D. P4
OPA1
m1 : p.(Tyr102Cys)
E. P5
OPA1
m1 : p.(Gly401Asp)
F. P6
OPA1
m1 : p.(Lys262*)
G. P7
OPA1
m1 : p.(Thr541del)
TMP SUP NAS INF TMP

12 **Supplementary Figure 4. Pedigree charts, fundus photographs, optical coherence tomographys and Humphrey visual fields in 7 patients with *OPA1*-mutations.** (A-G) Pedigree charts in 7 patients with *OPA1* mutations. (H-N) Fundus photographs showing temporal pallor in dominant optic atrophy, except P2. P2 showed generalized optic atrophy. (O-U) Spectral domain optic coherence tomography showed mainly temporal retinal nerve fiber layers thinning. The retinal nerve segmentation was not available in P2 due to nystagmus. (V-Z) Automated Humphrey visual field tests in patients with *OPA1*-dominant optic atrophy. All patients with *OPA1* mutations had temporal retinal nerve fiber layer defects except P2. The missense variant c.1240A>C:p.(Thr414Pro) is located in GTPase domain, and this variant may cause a dominant negative effect. The segmentation of retinal nerve fiber layers was not available due to multidirectional nystagmus. The ocular phenotypes such as visual acuity, degree of optic atrophy, the presence of nystagmus, were more severe in P2 than in others with *OPA1* mutations.

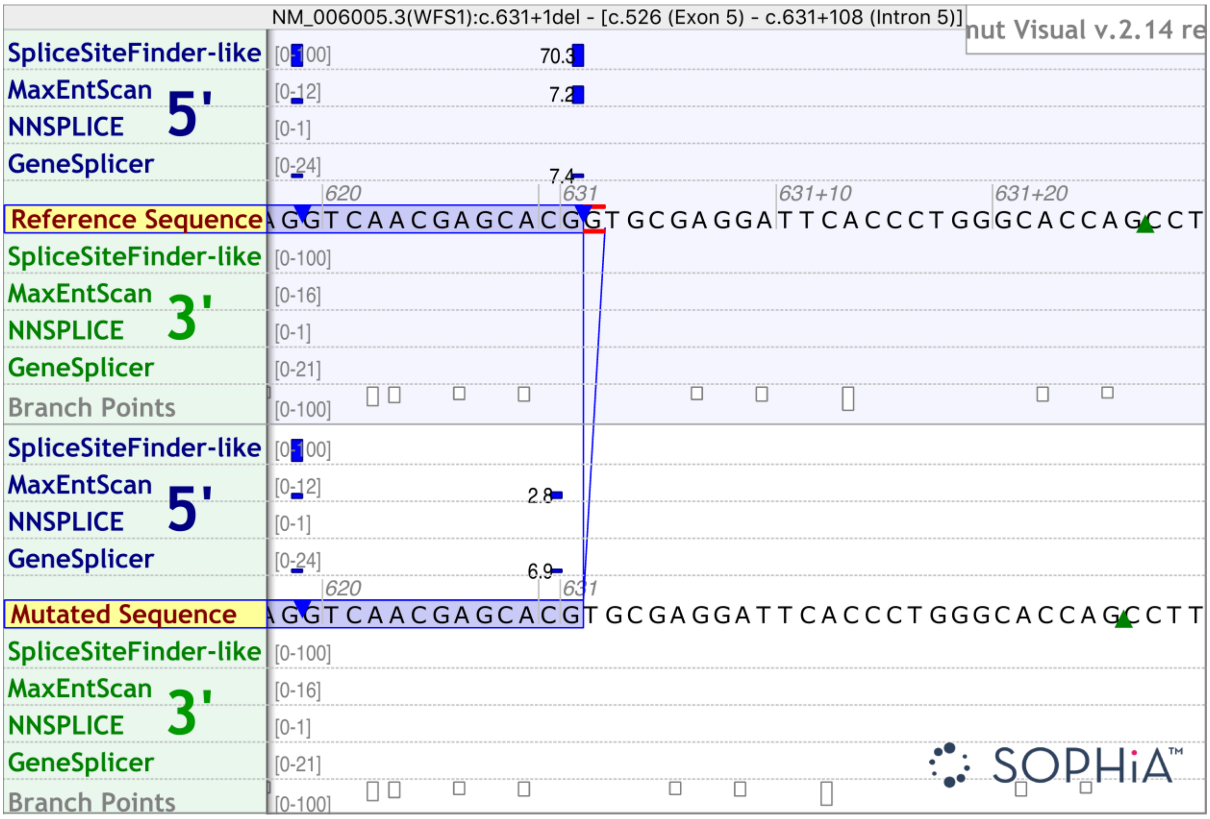


13 **Supplementary Figure 5.** The novel c.631+1del variant in *WFS1* gene causes disruption of canonical splicing donor sites. The splice site prediction was conducted through Alamut Visual v.2.14 (Interactive Biosoftware, Rouen, France).

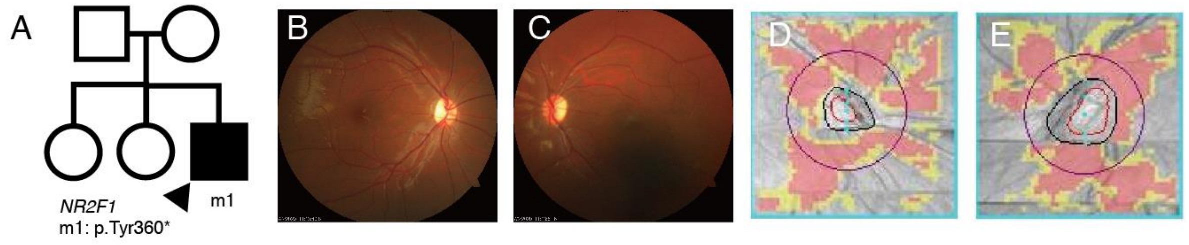


14 **Supplementary Figure 6. (P11) Pedigree chart, Fundus photographs and optic coherence tomography in Bosch Boonstra Schaaf optic atrophy syndrome.** This patient had latent nystagmus, and delayed development in early childhood. At the age of 25 years, he had normal intelligence and no other neurological symptoms. Best corrected visual acuity was 0.40 in the right eye and 0.52 in the left eye (logMAR). (A) A nonsense variant c.1080C>A:p.(Tyr360*) in *NR2F1* gene was detected in targeted NGS panel. (B, C) Fundus photographs showing diffuse loss of retinal nerve fiber layers. (D, E) Cirrus optic coherence tomography showing generalized thinning of peripapillary retinal nerve fiber layers.

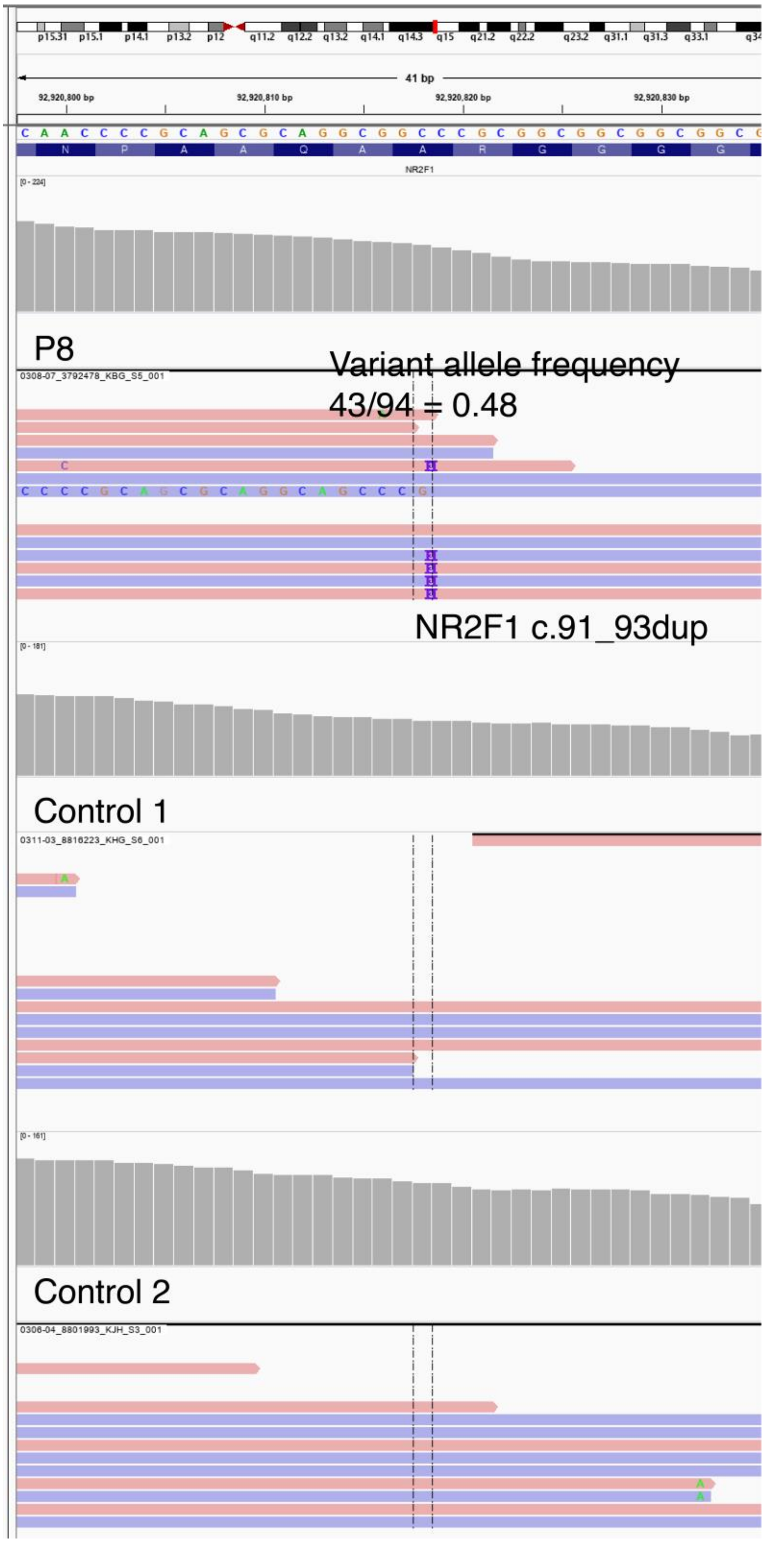


**Supplementary Figure 7.** The low coverage region of *NR2F1* exon 1 region (hg19 position- chr5:92,920,730~92,920,890) due to low-complex repetitive sequence. However, the targeted panel in our study covers this region sufficiently, and it can detect c.91_93dup:p(Arg31dup) variant (hg19 position-chr5: 92,920,820_92,920,822).

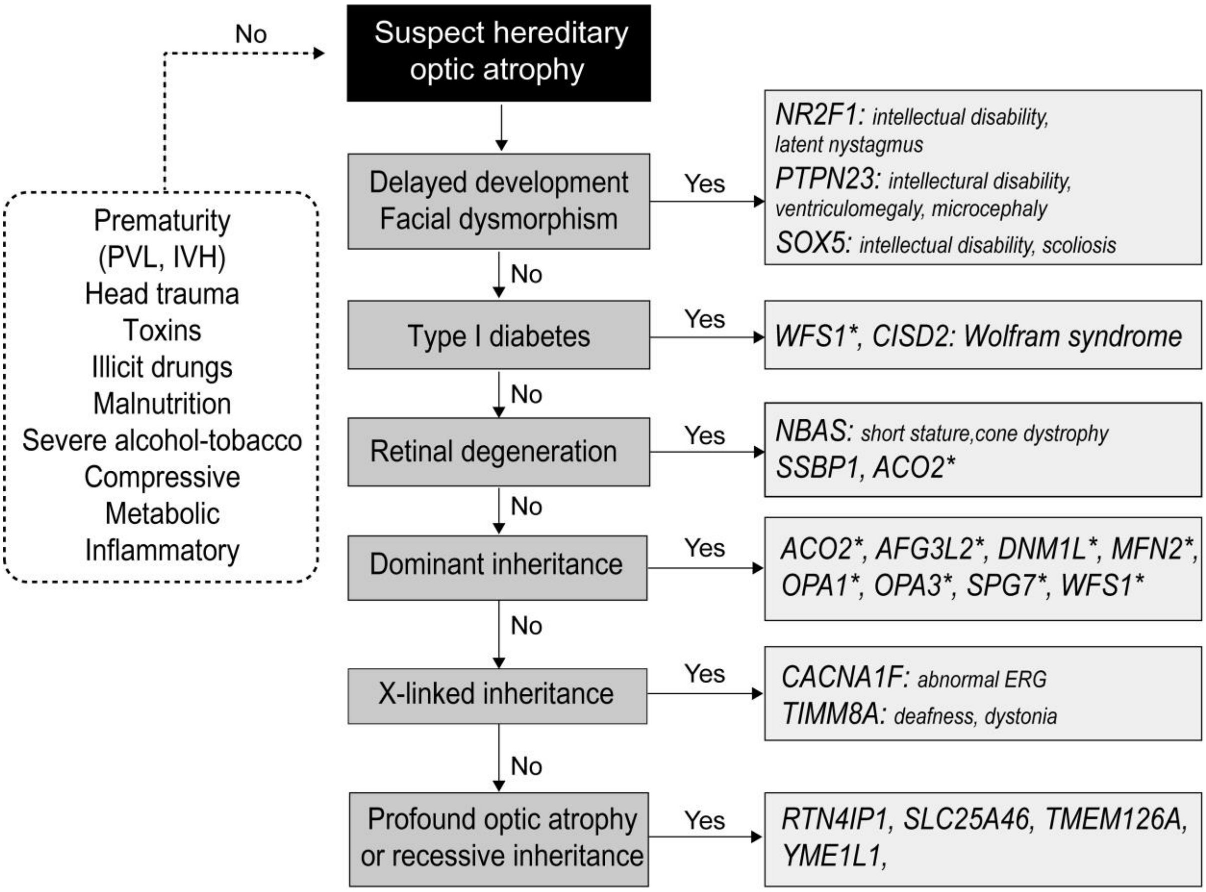


16 **Supplementary Figure 8.** Schematic representation of the diagnostic workflow for suspected hereditary optic atrophy. The asterisk (*) indicates that these genes can be inherited as either autosomal dominant or recessive. PVL = periventricular leukomalacia, IVH = intraventricular hemorrhage**.**

**References**


1. Rim JH, Lee ST, Gee HY, et al. Accuracy of Next-Generation Sequencing for Molecular Diagnosis in Patients With Infantile Nystagmus Syndrome. JAMA Ophthalmol 2017;135:1376-85.
2. Li H, Durbin R. Fast and accurate short read alignment with Burrows-Wheeler transform. Bioinformatics 2009;25:1754-60.
3. McKenna A, Hanna M, Banks E, et al. The Genome Analysis Toolkit: a MapReduce framework for analyzing next-generation DNA sequencing data. Genome Res 2010;20:1297-303.
4. Ye K, Schulz MH, Long Q, et al. Pindel: a pattern growth approach to detect break points of large deletions and medium sized insertions from paired-end short reads. Bioinformatics 2009;25:2865-71.
5. Chen X, Schulz-Trieglaff O, Shaw R, et al. Manta: rapid detection of structural variants and indels for germline and cancer sequencing applications. Bioinformatics 2016;32:1220-2.
6. Plagnol V, Curtis J, Epstein M, et al. A robust model for read count data in exome sequencing experiments and implications for copy number variant calling. Bioinformatics 2012;28:2747-54.
7. Kuilman T, Velds A, Kemper K, et al. CopywriteR: DNA copy number detection from off-target sequence data. Genome Biol 2015;16:49.
8. Stenson PD, Mort M, Ball EV, et al. The Human Gene Mutation Database: towards a comprehensive repository of inherited mutation data for medical research, genetic diagnosis and next-generation sequencing studies. Hum Genet 2017;136:665-77.
9. Kumar P, Henikoff S, Ng PC. Predicting the effects of coding non-synonymous variants on protein function using the SIFT algorithm. Nat Protoc 2009;4:1073-81.
10. Adzhubei IA, Schmidt S, Peshkin L, et al. A method and server for predicting damaging missense mutations. Nat Methods 2010;7:248-9.
11. Schwarz JM, Rödelsperger C, Schuelke M, Seelow D. MutationTaster evaluates disease-causing potential of sequence alterations. Nat Methods 2010;7:575-6.
12. Reva B, Antipin Y, Sander C. Predicting the functional impact of protein mutations: application to cancer genomics. Nucleic Acids Res 2011;39:e118.
13. Rentzsch P, Witten D, Cooper GM, et al. CADD: predicting the deleteriousness of variants throughout the human genome. Nucleic Acids Res 2019;47:D886-d94.
14. Pollard KS, Hubisz MJ, Rosenbloom KR, Siepel A. Detection of nonneutral substitution rates on mammalian phylogenies. Genome Res 2010;20:110-21.
15. Choi Y, Sims GE, Murphy S, et al. Predicting the functional effect of amino acid substitutions and indels. PLoS One 2012;7:e46688.
16. Shihab HA, Rogers MF, Gough J, et al. An integrative approach to predicting the functional effects of non-coding and coding sequence variation. Bioinformatics 2015;31:1536-43.
17. Ferlaino M, Rogers MF, Shihab HA, et al. An integrative approach to predicting the functional effects of small indels in non-coding regions of the human genome. BMC Bioinformatics 2017;18:442.
18. Xiong HY, Alipanahi B, Lee LJ, et al. RNA splicing. The human splicing code reveals new insights into the genetic determinants of disease. Science 2015;347:1254806.
19. Richards S, Aziz N, Bale S, et al. Standards and guidelines for the interpretation of sequence variants: a joint consensus recommendation of the American College of Medical Genetics and Genomics and the Association for Molecular Pathology. Genet Med 2015;17:405-24.
